# Metabarcoding quantifies differences in accumulation of ballast water borne biodiversity among three port systems in the United States

**DOI:** 10.1016/j.scitotenv.2020.141456

**Published:** 2020-08-03

**Authors:** John A. Darling, John Martinson, Katrina M. Pagenkopp Lohan, Katharine J. Carney, Erik Pilgrim, Aabir Banerji, Kimberly K. Holzer, Gregory M. Ruiz

**Affiliations:** a United States Environmental Protection Agency, Center for Environmental Measurement & Modeling, USA; b Smithsonian Environmental Research Center, Edgewater, MD 21037, USA; c United States Environmental Protection Agency, Center for Computational Toxicology & Exposure, USA

**Keywords:** Ballast water, Biosecurity, Metabarcoding, High throughput sequencing, Biodiversity, Zooplankton

## Abstract

Characterizing biodiversity conveyed in ships’ ballast water (BW), a global driver of biological invasions, is critically important for understanding risks posed by this key vector and establishing baselines to evaluate changes associated with BW management. Here we employ high throughput sequence (HTS) metabarcoding of the 18S small subunit rRNA to test for and quantify differences in the accumulation of BW-borne biodiversity among three distinct recipient port systems in the United States. These systems were located on three different coasts (Pacific, Gulf, and Atlantic) and chosen to reflect distinct tradepatterns and source port biogeography. Extensive sampling of BW tanks (n = 116) allowed detailed exploration of molecular diversity accumulation. Our results indicate that saturation of introduced zooplankton diversity may be achieved quickly, with fewer than 25 tanks needed to achieve 95% of the total extrapolated diversity, if sou rce biogeography is relatively limited. However, as predicted, port systems with much broader source geographies require more extensive sampling to estimate diversity, which continues to accumulate after sampling >100 discharges. The ability to identify BW sources using molecular indicators was also found to depend on the breadth of source biogeography and the extent to which sources had been sampled. These findings have implications both for the effort required to fully understand introduced diversity and for projecting risks associated with future changes to maritime traffic that may increase source biogeography for many recipient ports. Our data also suggest that molecular diversity may not decline significantly with BW age, indicating either that some organisms survive longer than recognized in previous studies or that nucleic acids from dead organisms persist in BW tanks. We present evidence for detection of potentially invasive species in arriving BW but discuss important caveats that preclude strong inferences regarding the presence of living representatives of these species in BW tanks.

## Graphical Abstract

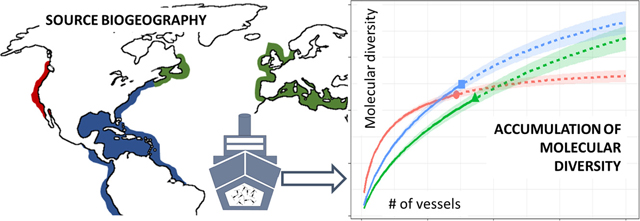

## 1. Introduction

For many centuries the movement of oceangoing vessels has reshaped global biogeography, resulting in dramatic homogenization of coastal marine biota (Darling and Carlton, 2018; Ricciardi, 2016; Seebens et al., 2016). Since the advent of modern shipping, transport of living organisms in ballast water (BW) has been one of the most efficient vectors of this biotic interchange and has been associated with numerous ecologically and economically devastating aquatic biological invasions (Carlton, 1985; Ricciardi and MacIsaac, 2000). Acknowledgment of the costly impacts of ballast water (BW)-borne species introductions has led to the development of multiple mechanisms aimed at risk mitigation, including the International Maritime Organization (IMO) 2004 International Convention for the Control and Management of Ship’s Ballast Water and Sediments, which entered into force in September 2017 (IMO, 2004). This international agreement mandates attainment of numerical organism discharge standards through treatment of BW, standards which have been similarly adopted by legal instruments governing BW transport at national levels, including the U. S. Environmental Protection Agency (USEPA) 2013 Vessel General Permit and the U.S. Coast Guard (USCG) 2012 final BW rule (USCG, 2012; USEPA, 2013).

Despite the emergence of these and other efforts to curb BW-borne invasions, BW transport is likely to continue contributing to the translocation of aquatic species in the foreseeable future (Gollasch et al., 2018). Full global compliance with discharge standards established in the IMO convention is not expected until 2024 (IMO, 2017), and challenges persist in evaluating how effective approved treatment systems will be under the broad range of conditions to which they might be exposed globally (Casas-Monroy et al., 2018; Hess-Erga et al., 2019; King and Tamburri, 2010). Additional uncertainty in the relationship between invasion rate and propagule pressure (Carlton et al., 2011; Wonham et al., 2013) means that the precise degree to which risk will be reduced by even full attainment of current numerical discharge standards remains unclear. Moreover, anticipated changes in international trade dynamics and associated shifts in shipping patterns may dramatically increase BW discharge to certain recipient regions in the future, elevating overall risk of invasion for those regions (Carney et al., 2017; Holzer et al., 2017). Thus, a better understanding of how vessels transport biodiversity in BW remains an important research need, both to assist in ongoing assessment of invasion risk and to determine how the global implementation of recently adopted standards will alter that risk.

Acquiring statistically robust data on biodiversity transported in ballast tanks has always been difficult due to the variety of organisms present, the broad biogeography sampled by uptake events, and logistical challenges associated with vessel and tank access (David and Perkovic, 2004). Nevertheless, considerable effort has resulted in growing appreciation for the biological diversity present in BW across geographic regions and taxonomic groups. The bulk of this effort has been conducted utilizing traditional morphological identification of organisms. Much of this work has thus focused on macroscopic zooplankton, which are typically amenable to this approach (Boltovskoy et al., 2011; Cordell et al., 2009; DiBacco et al., 2012), although some studies have attempted broader assessments including microbial taxa (Gollasch et al., 2002) or have specifically targeted groups such as phytoplankton or potentially toxic dinoflagellates (Roy et al., 2012; Villac and Kaczmarska, 2011). These and similar studies have consistently recognized a significant number of taxa of concern that either pose direct harm to humans and other animals via toxicity or are known to be non-indigenous to recipient regions. Taxonomic assignments in these morphological assessments have ranged broadly from species to phylum level, with significant limitations on identification of challenging taxonomic groups and many pre-adult stages (e.g. larvae and eggs) commonly found in BW.

To help overcome these limitations, a number of recent studies of BW-borne diversity have leveraged advances in high throughput sequencing (HTS) and bioinformatics to assess BW communities via DNA metabarcoding (Darling and Frederick, 2017; Rey et al., 2017). These approaches have substantially expanded the taxonomic scope and resolution of biodiversity assessments and, by decreasing the time and effort needed to generate data from large numbers of samples, have created opportunities for novel statistical analyses of BW diversity patterns. Assessments of bacterial and fungal communities, which have traditionally posed extreme challenges for conventional assessments, have benefited especially from these emerging tools (Hess-Erga et al., 2019). Recent studies have investigated diversity of bacterial communities across multiple samples entering ports both in the US (Brinkmeyer, 2016; Lymperopoulou and Dobbs, 2017) and internationally (Gerhard and Gunsch, 2019a; Johansson et al., 2017), while others have explored fungal (Gerhard and Gunsch, 2019b) and protistan diversity (Pagenkopp Lohan et al., 2016; Pagenkopp Lohan et al., 2017; Wu et al., 2017), including focal species with potential for negative human health impacts. Metabarcoding has also been employed to explore the impacts of BW treatments such as electrochlorination, UV exposure, and alkalinity on bacterial communities (Fujimoto et al., 2014; Petersen et al., 2019). Fewer studies have examined the utility of HTS-based methods for understanding diversity of zooplankton (Darling et al., 2018; Rey et al., 2019; Zaiko et al., 2015a; Zaiko et al., 2015b). Metabarcoding studies have often leveraged the considerable depth of diversity identified by HTS, with many hundreds to thousands of operational taxonomic units (OTUs) recovered per study, to investigate total diversity being delivered to recipient ports (Darling et al., 2018; Pagenkopp Lohan et al., 2017) or to attempt statistically rigorous classification of samples and develop biomarkers capable of identifying BW sources (Gerhard and Gunsch, 2019b), determining management status (Darling et al., 2018), or differentiating BW from port or open ocean water (Gerhard and Gunsch, 2019a).

While these studies have revealed substantial molecular diversity in BW samples, application of HTS to BW diversity assessment remains a developing field, and most studies have been limited to relatively small sample sizes; the largest recently published effort utilized 41 ballast samples from 4 international ports in Asia, Africa, and North America (Gerhard and Gunsch, 2019a). Exploring patterns of BW diversity accumulation at recipient ports will require substantially greater sampling efforts, comparable to earlier large-scale studies based on morphological taxonomy (Cordell et al., 2009; DiBacco et al., 2012; Gollasch et al., 2002). Here we look to extend this line of research by examining the molecular diversity entering three major US port systems (Chesapeake Bay, Galveston/Houston, Texas, and Valdez, Alaska) in 116 separate ballast tanks on vessels arriving from both domestic and international sources. Our primary aim in this work was to test the hypothesis that the biogeography of source ports, including geographically restricted versus extensive source regions, leads to quantifiable differences in the total diversity delivered to recipient port systems and, especially, the rate at which that diversity accumulates. We further explore differences in taxonomic composition of the communities delivered to recipient ports and examine the detection of OTUs of possible concern in biosecurity contexts. Our results confirm the value of metabarcoding for characterizing biodiversity present in ballast tanks and further suggest applications for uncovering BW-driven biotic exchange at regional and global scales. While application in regulatory contexts (e.g. testing compliance with numerical discharge standards) remains beyond the scope of existing molecular methods, this study demonstrates expanded utility of these tools to understand biodiversity flux, with important implications for risk assessment.

## 2. Methods

### 2.1. Sampling

**Fig. 1.**
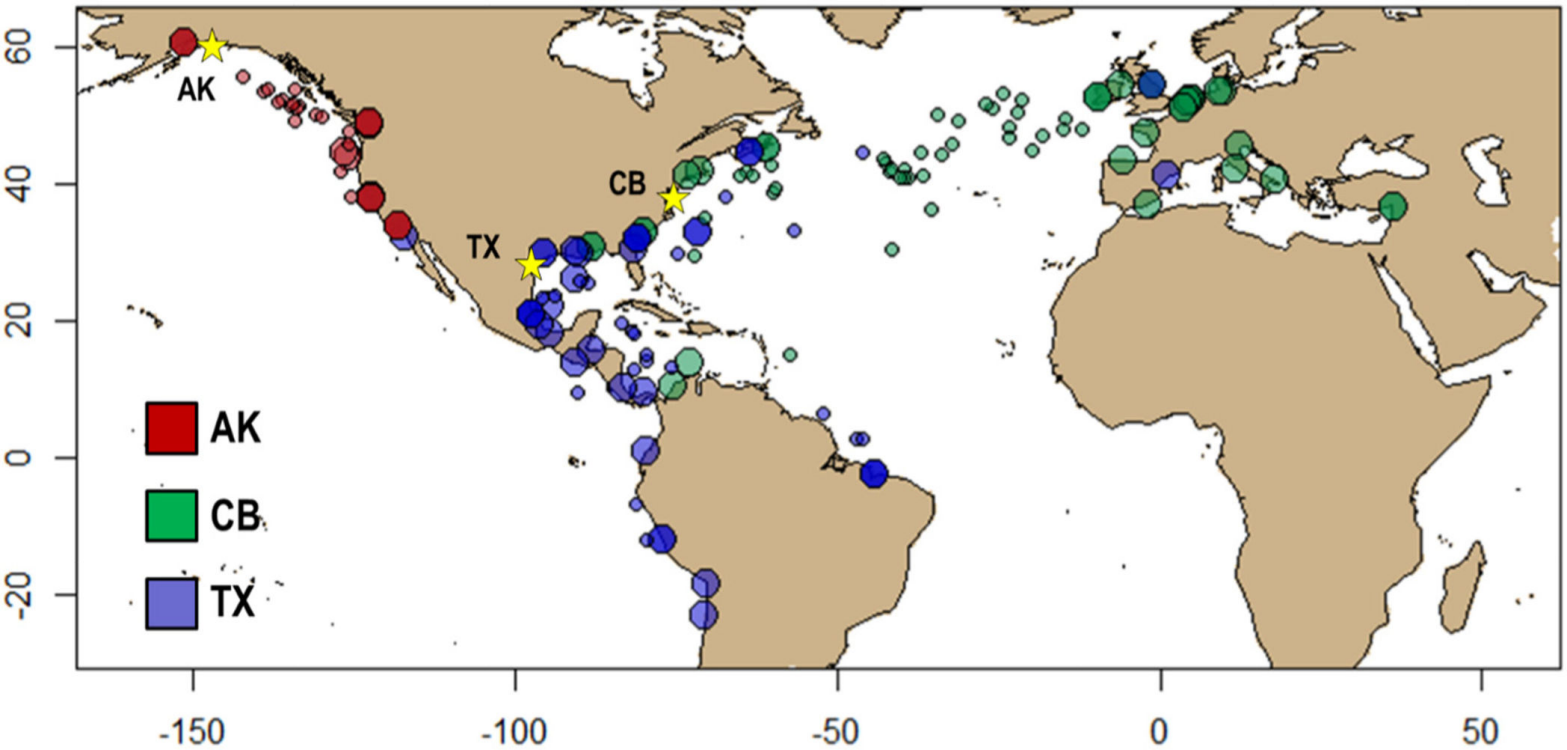
Distribution of source ports (large filled circles) for vessels entering Alaska (red), Chesapeake Bay (blue), and Texas (green). Destination ports are indicated with stars. Small filled circles indicate locations of ballast water exchange and are colored according to destination port. (For interpretation of the references to colour in this figure legend, the reader is referred to the web version of this article.)

Vessels were sampled between 2012 and 2014 on entry to three focal port systems, including the ports of Valdez, Alaska (AK), Texas City and Houston, Texas (TX), and Hampton Roads, Virginia and Baltimore, Maryland in the Chesapeake Bay (CB). Focal port systems were selected in three widely separated ocean basins in order to capture distinct trade patterns associated with vessel traffic as well as distinct source port bio-geographies. While vessels entering AK were engaged exclusively in coastwise voyages and derived from a limited source region, vessels entering CB and TX both represented a broad mix of overseas and domestic voyages from a large number of source ports and source regions. Both vessels conducting BWE and those carrying unmanaged BW were sampled; several vessels utilizing installed BW management systems were excluded from the current study as they represented only a very small sample size. The last port of call as well as BW source, age and management history was recorded for all vessels sampled (Fig. 1, Supplemental Table 1). In some cases, multiple unique samples of BW with different source and management histories were collected from multiple tanks on a single vessel. Where appropriate we therefore refer throughout the remainder of this paper to “tanks” or “samples” rather than “vessels” to reflect this distinction. BW sources were assigned to Large Marine Ecosystems (LME) (Sherman, 2005) based on maps obtained from the US National Oceanographic and Atmospheric Administration (David et al., 2015). Samples were obtained using a 35 μm mesh plankton net (50 μm diagonal dimension, consistent with the largest size class defined in IMO, USCG, and USEPA regulations) lowered from manholes on the deck and towed through the accessible water column to the surface. The tow depth varied between sites due to differences in tank configuration on different vessel types. Tow volumes were highest in Alaskan samples (mean: 0.92 ± 0.01 m^3^), and lower in Texas (mean: 0.45 ± 0.07 m^3^) and Chesapeake Bay samples (mean: 0.25 ± 0.02 m^3^). No corrections were made to account for variations in tow volume. Net and cod end were washed with filtered water and sample collected in a 125 mL sample bottle. Biomass was further concentrated in the laboratory by filtration through a 35 μm mesh sieve and preserved in 95% ethanol, then finally filtered through a 20 μm mesh filter and rinsed with ethanol prior to nucleic acids extraction.

### 2.2. Molecular methods

Detailed molecular methods, including reaction conditions and primer sequences, can be found in Darling et al. (2018) and associated supplemental materials. Samples were vacuum filtered through 0.8 μm polycarbonate filters and phenol-chloroform extracted. For negative controls a single sterile water extraction blank was run with every set of extractions and a separate filter blank was run on every day of extractions; these blanks were processed through remaining molecular and bioinformatics workflows along with standard negative PCR controls run alongside each set of DNA templates. A fragment of the small subunit (SSU or 18S) ribosomal RNA was amplified using primers described by (Blaxter et al., 1998). PCR products were cleaned, quantified, and normalized to 10 ng/μL before being prepared with adapters and multiplexing tags and run on an Illumina MiSeq with paired-end 300 bp kits. Initial PCRs were run with primers containing upstream adapters for secondary dual-indexing PCR. Dual-indexing amplicons were cleaned, quantified, and then normalized for addition to the MiSeq run. Index sequences were fed into the MiSeq before the run and were used by MiSeq software to assign sequences to each individual sample. All raw sequence reads have been deposited in Genbank, bioproject accession number PRJNA649942.

### 2.3. Bioinformatic analyses

A custom Perl script (all custom scripts are available from the authors on request) invoking cutAdapt (v. 1.12) was used to trim primer sequences from the 5′ end of both the forward and reverse FASTQ reads and sequences were merged using USEARCH (v. 9.2), resulting in 6.6 million pairs merged with a mean length of 347 bps. 964 reads with Phi X 174 contamination were removed and a cut-off of 350 bps was established for further processing; merged sequences longer than 350 bps were trimmed, and remaining sequences were filtered to a subset that had ≤1 expected errors (ee1). 6.1 M (92.1%) reads passed the length-only filtering and 3.9 M passed the ee1 filtering. The ee1 set was dereplicated, leaving 1.3 M unique reads, and these were clustered into OTUs at 97% identity, resulting in 1196 OTUs. The 6.1 M “length_only” filtered set of merged reads was then mapped to the OTU sequences to produce an OTU table; 5.3 M reads (88%) mapped to OTUs. To account for OTUs that appeared in negative controls we adopted the method described in Darling et al. (2018); this resulted in removal of 52 OTUs from the final table.

For taxonomic assignment OTU sequences were blasted (BLASTN) against a local copy of the NCBI nt database. Blast results were inspected and a bit score of ≥446, representing roughly a full length hit of 90% identity, was selected as a cut-off. A custom Perl script was used to download sequences with blast hits meeting the cut-off and their associated taxonomy records; taxonomy records were adjusted so that they had an equal number of standard levels, from kingdom to species, even if a given level was blank. USEARCH was then used to limit the reference sequences to unique instances, each with their corresponding adjusted taxonomies. Another Perl script then converted the USEARCH output into QIIME (v.1.9.1) compatible reference sequence and taxonomy files. The RDP classifier (v2.2), as implemented in QIIME, was run with the two reference files and otherwise default settings to assign taxonomy to the OTUs. For additional information on bioinformatic processing see Darling et al. (2018).

### 2.4. Statistical analyses

All statistical analyses were conducted in the *R* statistical computing environment, v. 3.5.3 (Team, 2017). To explore our ability to identify sources of ballast based on OTU diversity data, ordination analysis were conducted in the *vegan* package (Oksanen et al., 2016) using log-transformed count data after rarefaction to the median sample size. Non-metric dimensional scaling (NMDS) was implemented in two dimensions using Bray-Curtis distances. Statistical significance of clusters in unconstrained ordinations was assessed by testing partitions of sums of squares in the *adonis* function with 1000 permutations (Anderson, 2001). Tests of dispersion around cluster centroids for the three destination ports were conducted using the *betadisper* function in *vegan*, with significance assessed by ANOVA. The relationship between NMDS axis 2 (in ordinations including all vessels) and latitude of ballast water uptake (either initial uptake for unmanaged vessels or location of ballast water exchange (BWE) for managed vessels) was further assessed by linear regression.

The accumulation of molecular diversity in the three recipient ports was investigated using the incidence frequency method of (Chao et al., 2014) implemented in the *iNEXT* package (Hsieh et al., 2016). The same package was utilized to estimate sampling completeness and to determine the number of vessels that would have to be sampled to obtain 50%, 90%, 95%, and 99% coverage of the extrapolated OTU pool for each recipient port. To estimate contributions of different spatial scales to measured gamma diversity the *adipart* function of the *vegan* package was used to additively partition diversity among individual vessels, among source ports within LME, and among LME. To examine the association of diversity with voyage length, linear regression was employed to test for relationships between diversity and ballast water age, using extrapolated species richness (Chao’s estimate) generated by the *specpool* function in *vegan*.

## 3. Results

### 3.1. Summary statistics

Of the 116 tanks included in the dataset (see Fig. 1 and Table S1 for details), 36 entered AK from 12 different source ports distributed across 2 source LMEs; 42 entered CB from 26 ports across 10 LMEs; and 38 entered TX from 27 ports across 10 LMEs. The source region for AK had almost no overlap with source regions for CB and TX, which overlapped considerably in Central and North America. Overall, 75 of 116 tanks (65%) underwent BWE, including 17 entering AK (47%), 36 entering CB (86%), and 22 entering TX (56%). Average voyage length was 13.2 days (range of 1 to 48 days) and average age of ballast on discharge (when considering BWE) was 8.3 days (range of 1 to 28 days).

The average read count across samples was 27,076 (range of 8461 to 56,750 reads). Sequencing depth was similar across the three recipient ports, with mean read counts of 22,551, 29,685, and 28,479 for AK, CB, and TX, respectively. Rarefaction curves indicated that sequence depths greater than 5000 were sufficient to capture greater than 95% of observed OTUs in almost all samples (not shown). Mean observed OTU richness ranged widely from 6 to 172 (overall mean = 63.4), and Chao’s estimated richness ranged from 7 to 239 (mean = 81.1). Diversity differed significantly across recipient ports; mean observed and estimated richness for AK were 108.4 and 139.1, respectively, considerably and significantly higher than values for CB (36.8 and 48.9) and TX (50.2 and 61.7). There was no significant relationship between read count and diversity measures, again consistent with sequence coverage being sufficient to capture the vast majority of diversity present in samples. In total, 1144 OTUs were identified, 629 (55%) of which were assigned taxonomic identity at the species level. Remaining OTUs were either assigned identities at the genus level or above or were not assigned to any known taxonomic group. Multiple OTUs were sometimes assigned to the same species, such that a total of 484 species were identified across the entire dataset, with the remaining 515 OTUs being assigned identities above species level or not at all.

### 3.2. Geographic patterns of biodiversity and accumulation of OTUs in BW discharges

**Fig. 2.**
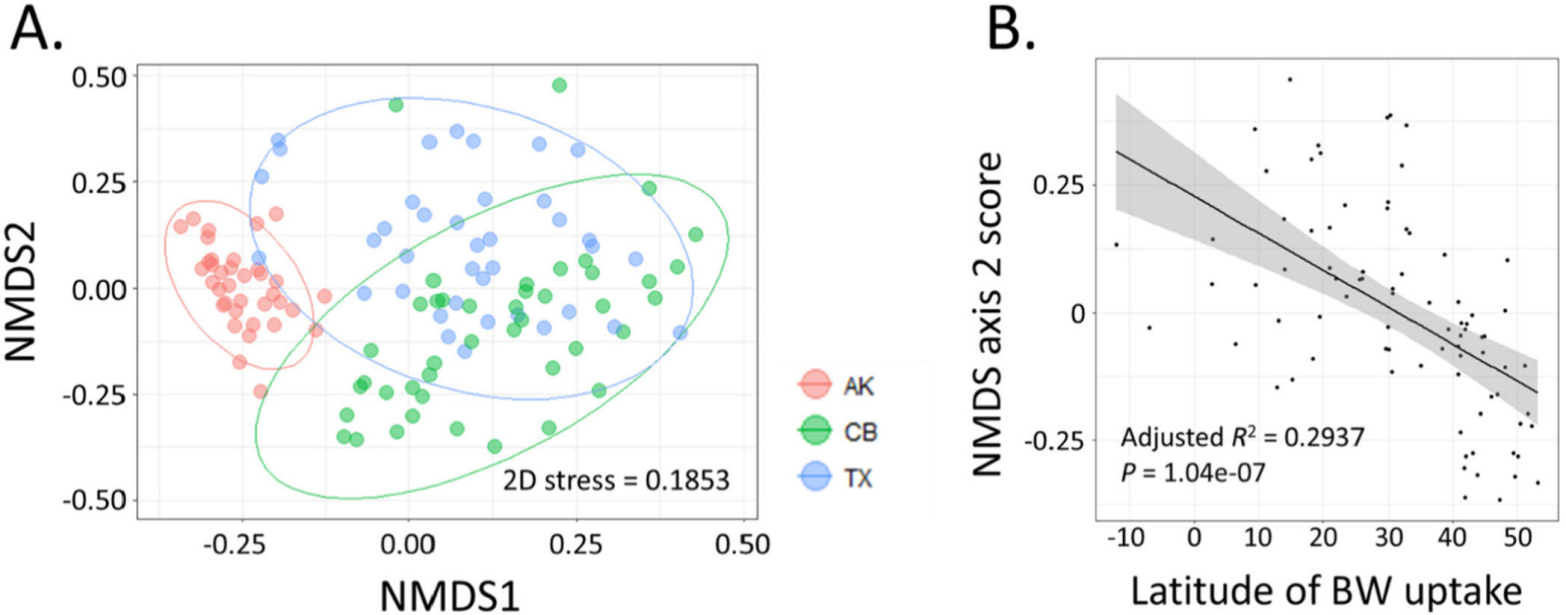
A. NMDS ordination of all samples. Clustering of vessels by destination port is significant (P < 0.001). B. Regression of NMDS axis 2 score against latitude of ballast water uptake for vessels entering CB and TX. Regression is significant also when analyzing unmanaged vessels (adjusted R^2^ = 0.2030, P = 0.0203) or managed vessels (adjusted R^2^ = 0.2997, P = 4.3178e−06) separately.

Destination port was a significant but weak predictor of clustering in unconstrained ordination by NMDS (*R*^2^ = 0.0341, P < 0.001). NMDS axis 1 largely separated tanks entering AK from those entering CB and TX, and NMDS axis 2 separated CB from TX (Fig. 2A); axis 2 scores significantly correlated with the latitude of BW uptake (Fig. 2B, adjusted *R*^2^ = 0.2937, P = 1.04e−7). However, samples could not be clearly distinguished at finer geographic scales. NMDS ordination of all unmanaged tanks entering AK and TX revealed clear clustering of AK voyages, but failed to uncover significant signatures of source geography among TX samples even at the level of LME (P = 0.1849, see Fig. S1). BWE similarly had weak but significant effects on clustering (Fig. S2).

**Fig. 3.**
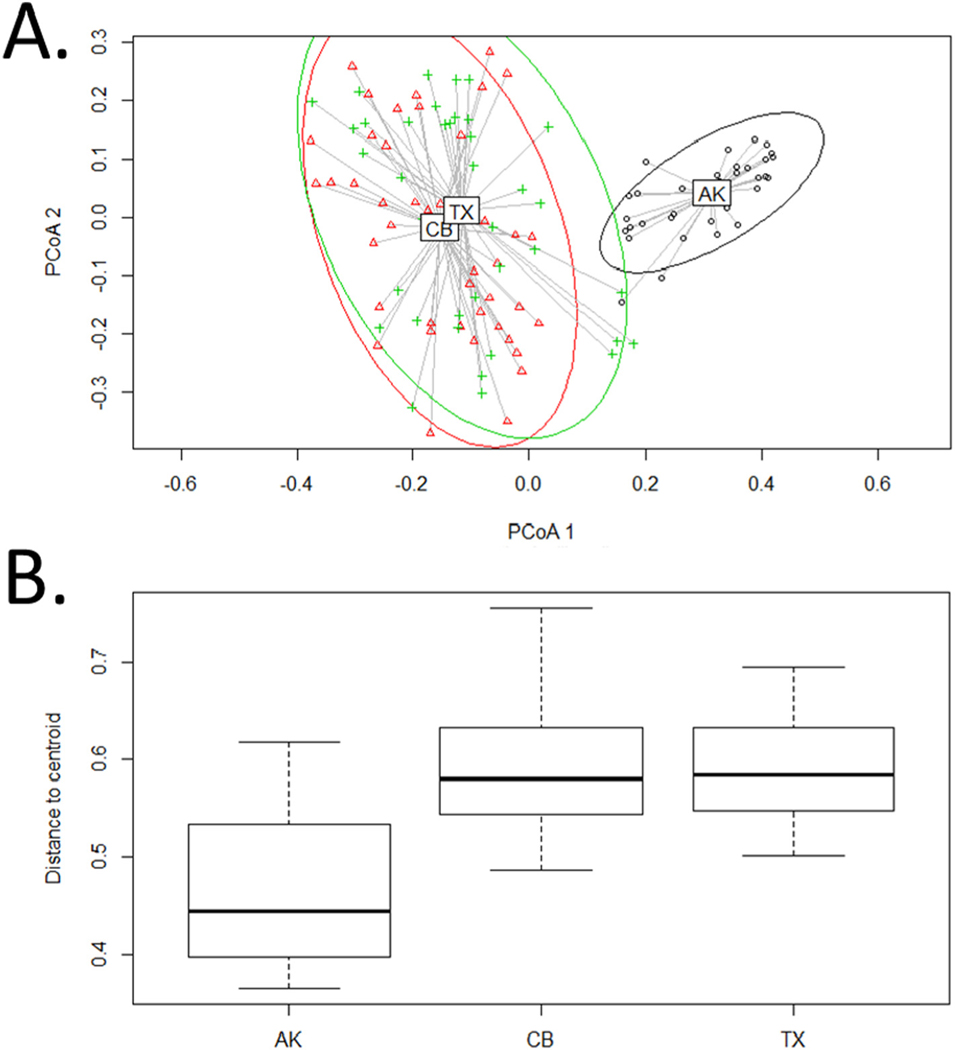
A. Principle components analysis of dispersion; individual points represent samples, cluster centroids are identified with port name abbreviations. B. Mean distance to cluster centroid for the three port systems. Vessels entering CB and TX are significantly more dispersed than those entering AK (P = 5.187e−15), consistent with those vessels sampling a much higher source diversity pool.

**Fig. 4.**
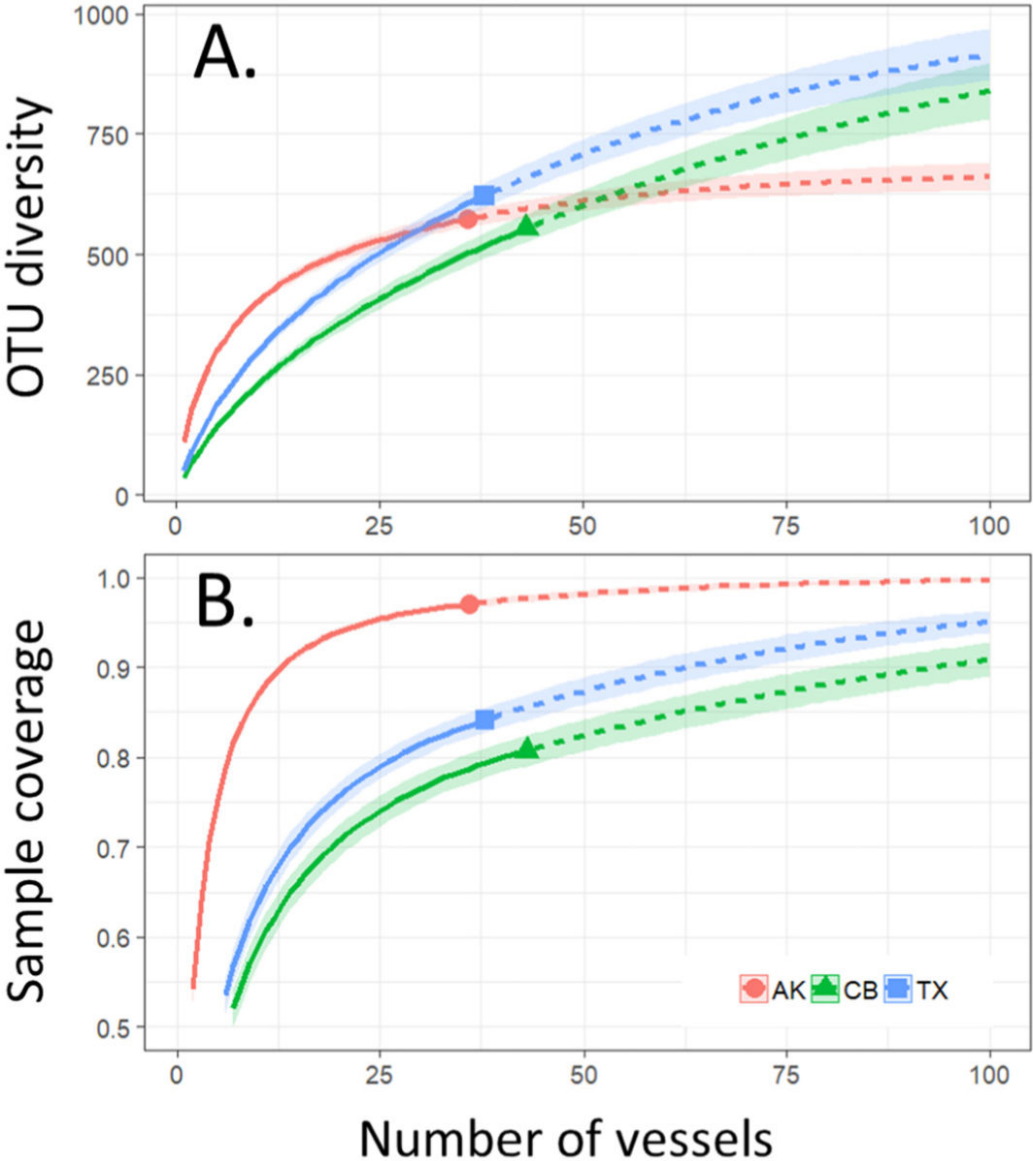
A) OTU accumulation curves for vessels entering AK (red), CB (green), and TX (blue). Chao’s extrapolated estimates for the total OTU pool for each destination port were 672 ± 42 for AK, 1099 ± 176 for CB, and 1050 ± 134 for TX. B) Sample completeness curves, showing fractional sample coverage (y axis) versus number of sampling units. Solid lines show observed data while dashed lines represent model extrapolations.

**Fig. 5.**
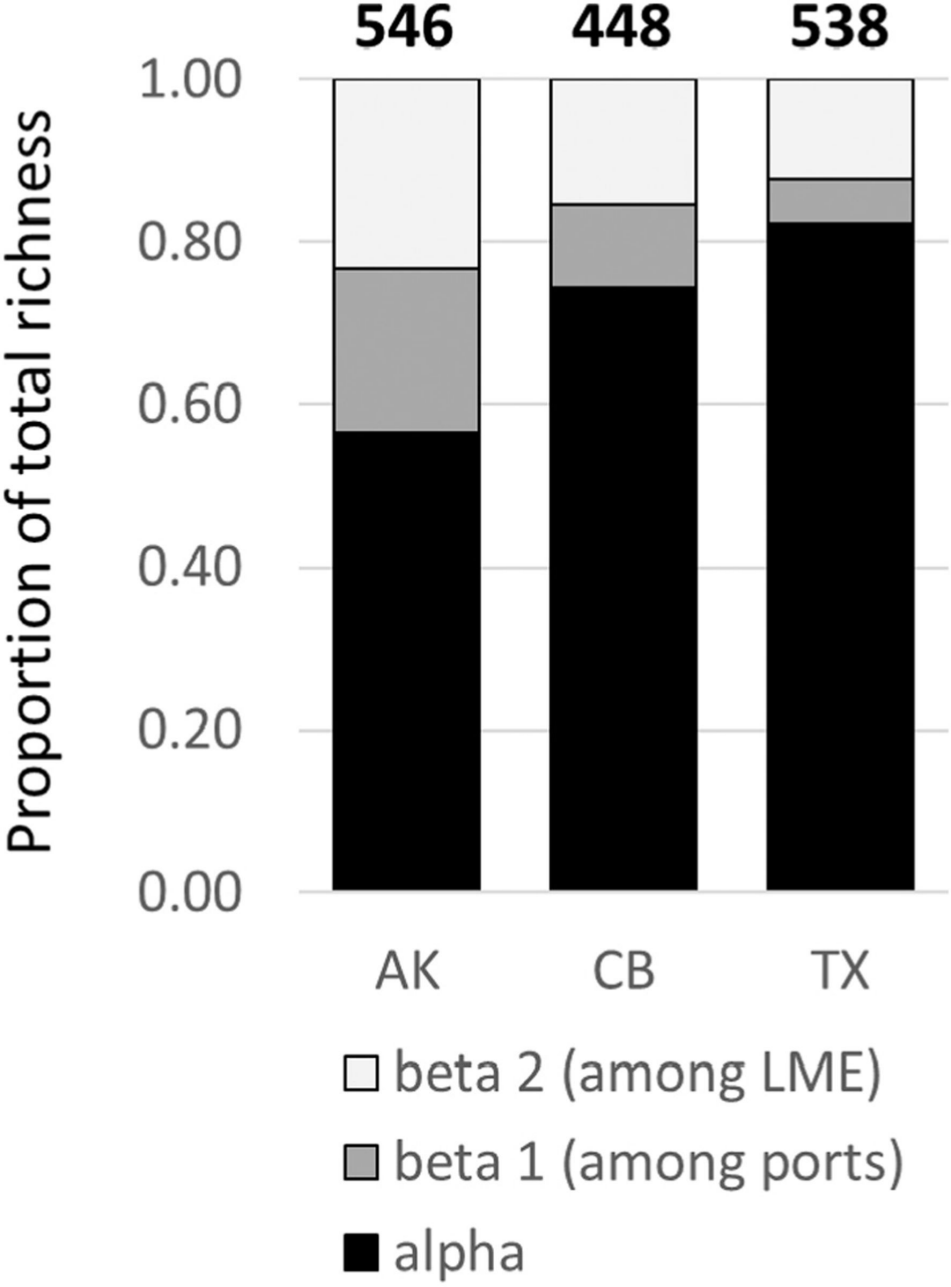
Proportional contributions of alpha diversity and beta diversity (beta 1 = among ports within LME, beta 2 = among LME) to gamma diversity for each of the three recipient ports. Total gamma diversity for each recipient port is indicated above the bar.

Tests of dispersion indicated that AK tanks are significantly less distinct from each other than are tanks entering CB or TX (Fig. 3). OTU accumulation curves similarly revealed much earlier saturation for samples entering AK (Fig. 4A). Extrapolated richness (Chao’s estimate) for the overall OTU pool entering the three ports was 672 ± 42 for AK, 1099 ± 176 for CB, and 1050 ± 134 for TX. Completeness curves showed that over 95% of the diversity entering AK was captured by the existing 36 samples (Fig. 4B). In contrast, existing samples captured only approximately 80% of estimated total diversity entering CB, and approximately 85% of diversity entering TX. A 95% sampling completeness was predicted after sampling only 23 tanks entering AK, while the same level of completeness would require an estimated 146 tanks entering CB and 99 entering TX (Table 1). Consistent with these analyses, additive partitioning of variance showed considerably higher proportional contributions of alpha diversity (within samples) to overall gamma diversity for CB and TX, compared to AK (Fig. 5). OTU accumulation curves were not substantially different between tanks conducting BWE and those that did not manage their ballast (Fig. S3).

### 3.3. BW age and diversity

**Fig. 6.**
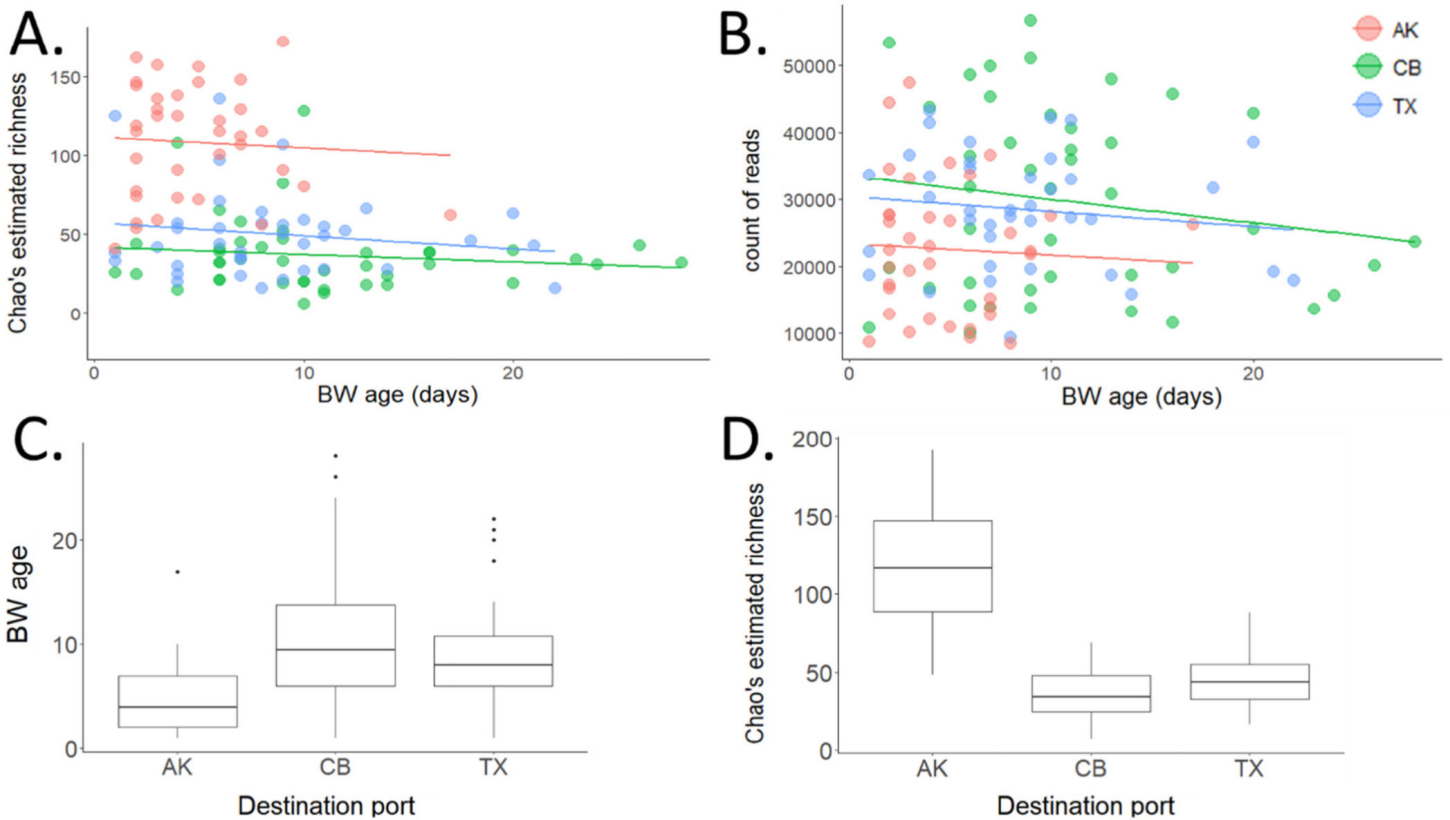
Relationship of estimated richness (A) and sequence read count (B) to ballast water age (in days). Although all trends are negative, slopes are not significantly different from zero for any destination port. Correlations were explored independently for each destination port since different ports exhibit significant differences in both average voyage length (C, P = 1.9e−6) and average diversity (D, P = 8.2e−15).

Ballast water age was not found to correlate with either diversity (Chao’s estimated OTU richness) or read count, our best proxy measure for abundance. Although overall OTU richness did decline significantly with increasing BW age across all samples, this was because samples entering AK harbored both significantly higher average OTU richness per tank and significantly lower average BW age due to shorter voyage lengths (Fig. 6). When each recipient port was considered separately both OTU richness and read count decreased with increasing BW age in all cases, but none of these relationships was significant.

### 3.4. Taxonomic assessments and OTUs of concern

**Fig. 7.**
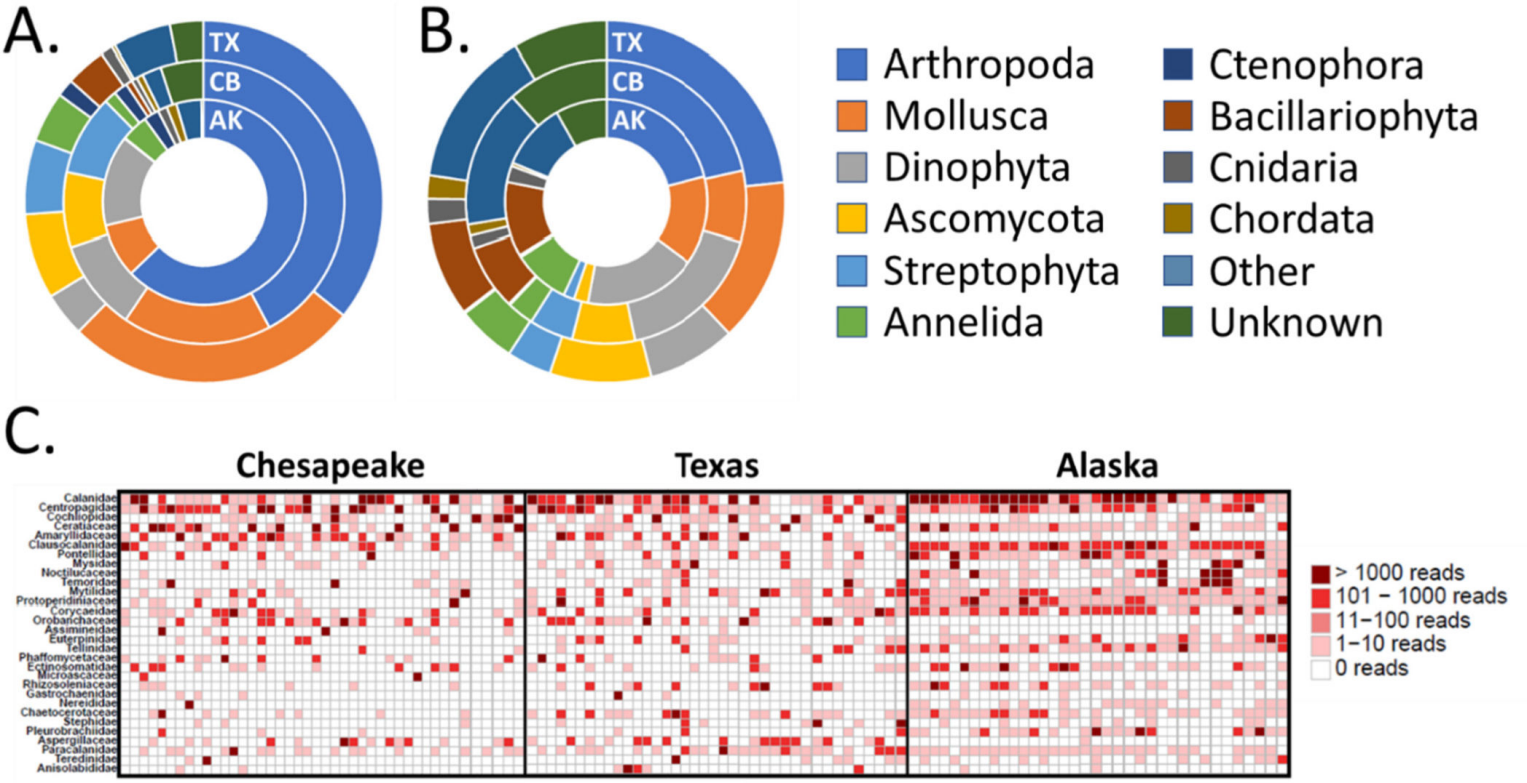
Distribution across recipient ports of (A) phyla based on sequence count, (B) phyla based on number of OTUs observed in each phylum, and (C) top 30 most abundant families. For both A and B only phyla with >1% total overall abundance are shown, all others are grouped as “other.” Solid vertical lines separate samples entering different recipient ports.

Consistent with observations of significant differences in NMDS ordinations, taxonomic groups were differentially distributed across the three recipient ports, both at the phylum and family level (Fig. 7). In particular, tanks entering AK showed considerably higher proportions of arthropods, especially copepods in the families Calanidae, Clausocalanidae, and Corycaeidae, whereas CB and TX samples showed greater dominance of mollusks and considerably higher proportions of ascomycota fungi. We identified 29 OTUs representing 23 species as “invasive,” considered here simply to mean having a known history of invasion according to either the WRiMS or NEMESIS databases (Table S2). Level of confidence in species level assignments for these OTUs varied considerably, and not all assignments were unambiguously supported by multiple analyses (see discussion and notes in Table S2). These “OTUs of possible concern” ranged from extremely rare (2 reads from a single voyage) to nearly ubiquitous (over 50,000 reads spread across 31 voyages entering all three ports).

## 4. Discussion

HTS-based approaches such as those employed in the current study are generally expected to provide a fuller account of extant biodiversity, in part because they are more likely to capture the “hidden and dark diversity” associated with taxa that are locally rare or impossible to evaluate in morphological studies (Partel, 2014). As illustrated in Fig. 7, analysis of the 18S locus enabled us to capture diversity from an expansive range of eukaryotic taxa, resulting in a diverse assemblage much broader than that recognized by all but the most ambitious morphological studies (see, e.g. Boltovskoy et al., 2011; Cordell et al., 2009; DiBacco et al., 2012; Gollasch et al., 2002). Primers targeting this region tend to amplify broadly across eukaryotic taxa, including metazoans, plants, diatoms, fungi, and protistan species, and can often result in extremely high OTU diversity as observed in the current study (Clarke et al., 2017)

While OTU counts across all samples provide some measure of the overall diversity being conveyed into the three recipient ports, OTU accumulation curves suggest that in the case of both CB and TX the total diversity being introduced to these ports has been under-sampled (Fig. 4, Table 1). Well over 95% of the extrapolated OTU richness entering AK is accounted for by the 36 samples in our dataset. However, the effort expended to sample CB and TX arrivals could only account for approximately 80% and 85%, respectively, of the projected total diversity, which exceeds 1000 OTUs in both cases. Extrapolated OTU accumulation curves suggest that much more extensive sampling would be required to fully capture the diversity entering these two ports—146 and 99 tanks to achieve 95% sample coverage for CB and TX, respectively (Table 1).

To our knowledge analogous attempts have not been previously made to estimate overall diversity conveyed to recipient ports or to uncover the variables driving patterns of accumulation. In one study based on a subset of the samples investigated here Pagenkopp Lohan et al. (2017) described OTU accumulation curves for protistan diversity, but more limited sampling and extremely high molecular diversity precluded extrapolation of total expected diversity or examination of the variables determining the rate at which it is delivered. Our results highlight the value of metabarcoding approaches for estimating the size of BW-borne species pools in a much more quantitative manner than has been available to date. This capability is likely due to the detection of a substantial fraction of hidden or dark taxa that were previously inaccessible to morphological analysis, and it is likely to further improve with future advances in sequencing technology, reference databases, and bioinformatic processing.

Apart from demonstrating the value of metabarcoding for estimating sampling efforts needed to characterize overall species pools, our study also confirms the expected importance of source biogeography in determining the total diversity being delivered to recipient ports and the rate at which it is delivered. The diversity introduced to recipient ports is expected to scale with both the number of vessels arriving and the source biogeography being sampled by those vessels (Blackburn et al., 2019). The dramatic differences observed between AK, which exhibits a relatively steep OTU accumulation curve, and CB and TX, which both exhibit shallower curves plateauing at significantly higher final extrapolated diversity, clearly reflect differences in source biogeography. Vessels entering AK all engage in intracoastal traffic and derive BW from source ports in the northeast Pacific, and therefore draw from limited sources distributed across only 2 LMEs. Vessels entering CB and TX, in contrast, each draw from over 10 LMEs spread across multiple ocean basins. These differences in source biodiversity manifest in significant differences in dispersion around cluster centroids in ordination of samples (Fig. 3).

The more thorough sampling of AK sources allows clearer resolution of differences in diversity between source regions and even source ports, with implications for the identification of BW sources. A previous study focusing only on tanks entering AK observed a strong signal of source port in ordinations of vessels carrying unmanaged ballast, indicating that source diversity could be resolved in those cases where BWE had not diluted the source signal (Darling et al., 2018). Here we observed significant differences in the source diversity being sampled by the three recipient systems, but only at very broad geographic scales. Latitude of source port did have a significant effect on BW diversity. Vessels entering AK could generally be distinguished from those entering CB and TX, but those entering the latter two ports exhibited largely overlapping clusters in NMDS ordinations (Fig. 2). More importantly, unlike previous analyses of AK arrivals, we were unable to identify a signal of source biodiversity for unmanaged BW entering TX, even at the level of source LME (Fig. S1). This lack of discernment is reflected in the relative contributions of alpha and beta diversity across the three recipient systems (Fig. 5). A considerably higher proportion of overall gamma diversity could be attributed to differences among ports and among LME for voyages entering AK compared to those entering CB and TX. For the former, it appears that source diversity has been sampled thoroughly enough to enable detection of clear signals of regional differentiation, whereas for CB and TX the source biogeography is sufficiently broad that the vast majority of overall diversity is attributable only at the alpha level and it is difficult to identify patterns of regional diversity. Other molecular studies of BW diversity have demonstrated similarly coarse resolution in identifying sources (Gerhard and Gunsch, 2019a; Lymperopoulou and Dobbs, 2017).

The patterns of diversity accumulation observed here have implications for projections of future risk associated with BW-borne introductions. While increased vessel traffic along existing trade routes is certain to increase propagule pressure, saturation of the introduced species pool is likely to occur with relatively few voyages if the sampled source region is constrained (as observed for AK in the current study). However, addition of new source regions is very likely to dramatically increase the diversity of introduced taxa. The importance of this “colonization pressure” (the number of species introduced to an area) in determining non-native species richness has been widely recognized (Blackburn et al., 2019; Lockwood et al., 2009). Results presented here suggest that expanding the biogeographic source region of a recipient port will lead to predictable increases in colonization pressure, with associated increases in the risk of introduction of ecologically and economically deleterious species. This is of particular concern given likely future shifts in maritime traffic as a result of changing global markets and the opening of new shipping routes (Carney et al., 2017; Miller and Ruiz, 2014; Sardain et al., 2019). These changes will almost certainly expand the source biogeography for some recipient ports, exposing them to increases in introduced biodiversity and potentially elevating risk of invasion. Changes to shipping traffic occurring between the time of sampling (2012–2014) and the present may already have had predictable impacts on patterns of biodiversity transfer into US ports. Examination of data on BW entering the three port systems suggests that while traffic into AK has been stable over the last decade, there have been increases in arrivals from South and East Asia to both CB and TX, primarily as a result of changes in global fossil fuels markets (National Ballast Information Clearinghouse, 2020; data not shown). Based on results obtained here, contemporary sampling of ballast entering these two ports would be expected to yield higher diversity than the baseline observed in 2012–2014 as a result of expanded source port geography. The demonstrated importance of source diversity also suggests that temporal shifts in environmental conditions driven by climate change or other anthropogenic stressors may similarly affect future patterns of BW-borne diversity transfers by altering the composition and structure of source port biological communities.

Previous studies have reported significant negative correlations between BW age and biotic assemblages. In an analysis of 380 BW tanks being discharged into Puget Sound in the northeast Pacific, Cordell et al. (2009) observed a significant reduction in zooplankton abundance with increasing BW age for both trans-Pacific and coastwise voyages and suggested that higher densities delivered by ships in coastwise trade may reflect in part their shorter transit times compared to overseas voyages. While that study did not investigate changes in taxonomic diversity, a number of other studies employing morphological analysis of both phyto- and zooplankton have described dramatic declines in both abundance and richness during the course of individual voyages of varying lengths (Gollasch et al., 2000a; Gollasch et al., 2000b; Olenin et al., 2000). Using molecular approaches, Johansson et al. (2017) similarly found that overall taxonomic richness of microbial communities assessed by 16S sequencing declined significantly over time during three separate transits of the Canadian arctic. Another HTS-based examination of metazoan diversity across 39 BW samples also found significant negative correlation of BW age with molecular diversity (Rey et al., 2019). In contrast, while we did observe declines in both OTU diversity and sequence read abundance consistent with these previous studies, correlations were weak and neither of these relationships was statistically significant (Fig. 6). There may be multiple reasons for this discrepancy. The breadth of taxonomic coverage made available by targeting the 18S locus has enabled us to include taxa that may be less susceptible to decline with lengthy voyages; indeed, previous work has shown that some taxa may even increase in abundance with BW age (Zaiko et al., 2015a; Zaiko et al., 2015b). It is also very likely that molecular methods are consistently more effective at detection of organisms at low abundance and of life-stages that are immature or difficult to identify morphologically. Of greater concern is the possible persistence of DNA from deceased organisms (see also below), the extent of which has yet to be quantified in the context of BW (Zaiko et al., 2015b) and which would clearly attenuate any signal of decline in either abundance or diversity. It is worth noting that across all voyages we did observe a significant decline in both read count and Chao’s richness with age; however, this pattern was driven entirely by a spurious correlation caused by elevated per sample diversity entering AK on relatively brief coastwise transits (Fig. 6C and D). Unlike direct assessments of changes in diversity over the course of individual voyages, correlation analyses such as ours are susceptible to such spurious signals and should therefore be interpreted with caution.

Even in the absence of taxonomic assignments, HTS data allow investigation of diversity accumulation patterns, assessments of diversity across samples, and efforts to identify biological indicators associated with specific conditions (e.g., BW sources or management status). These “taxonomy-free” approaches are gaining traction in various biomonitoring contexts and may represent a significant added value of HTS-based methods (Apotheloz-Perret-Gentil et al., 2017; Cordier et al., 2018; Mächler et al., 2020). Nevertheless, for many applications the identities of taxa present in samples are obviously of critical importance. This is particularly true in biosecurity contexts, where the detection of known or suspected invasive species may trigger management action.

Our analyses indicate the presence of 29 “OTUs of possible concern” that have been assigned to 23 species recognized as invasive in at least some part of their global range (Table S2). While these OTUs offer molecular evidence that species of biosecurity concern may be present in BW entering US recipient ports, there are important caveats to this message. Specifically, as discussed above, the presence of DNA from a particular species is not necessarily indicative of the presence of living organisms (Pochon et al., 2017). Additionally, taxonomic assignments can be uncertain and typically require assessment beyond those available in standard bioinformatics pipelines to determine confidence in species detections (Alberdi et al., 2018; Deiner et al., 2017). These considerations justify caution in interpreting detection of species of concern in standard HTS datasets (Darling et al., 2020). In some cases (e.g. detection of the mysid *Neomysis integer*), the observation of very high read counts on multiple voyages argues strongly for the presence of living organisms in these tanks. However, while *N*. *integer* represents the most likely assignment for OTUs 19 and 862, nearly perfect matches are also observed for *N. americana*, a North American native. Ambiguity in reference sequence databases—possibly combined with the inability of 18S data to resolve species level difference within this genus—therefore preclude strong inferences regarding the presence of *N*. *integer* in these BW samples. In other cases, taxonomic assignments may be supported by distributional evidence, allowing for stronger inferences. For instance, the invasive polychaete *Pseudopolydora paucibranchiata,* observed from 16 different voyages entering AK, is a known invasive species from the source region of these voyages on the Pacific coast of North America. Nevertheless, in the absence of more rigorous assessments of these taxonomic assignments we recommend that the observation of OTUs listed in Table S1 be taken as very preliminary indication of the presence of these species in BW entering US ports, providing a potential early warning to guide further analysis and detection.

These considerations highlight the challenges associated with developing molecular methods for application in contexts such as testing compliance with numerical discharge standards (Darling and Frederick, 2017; Rey et al., 2017). Nevertheless, HTS-based methods may have considerable value for research with direct implications for decision-making within existing regulatory frameworks. The IMO convention provides for permanent exemptions from BW management requirements when uptake and discharge of BW occur at the “same location” (IMO, 2004). This is generally intended to prevent over-regulation of transits occuring between locations that share common biota and therefore pose relatively low risk of invasion. However, the degree to which biota are shared across political, environmental, hydro-logical, or even biogeographical boundaries may vary dramatically (David et al., 2013). HTS-based assessments of biodiversity may prove a powerful tool in defining the “same location” concept by providing extensive data to formally evaluate biodiversity present in source and recipient ports, as well as the component of that diversity conveyed in BW.

## 5. Conclusions

HTS-based metabarcoding represents a potentially powerful tool for understanding patterns of biodiversity transported in BW. Greater access to hidden and dark diversity through these approaches allows more comprehensive assessments of overall diversity, enabling quantitative examination of species pools and the patterns with which they accumulate in ways previously unavailable in studies based on morphological methods. Our results demonstrate these benefits, quantifying differences in the total diversity delivered to recipient port systems and confirming the important role of trade patterns and source biogeography in determining the rate at which such diversity accumulates. Of considerable importance is the fact that such patterns can be discerned through statistical analyses of raw unassigned OTUs, thus avoiding widely recognized challenges associated with taxonomic identification and providing a robust method to evaluate changes in space and time as well as responses to BW management. While metabarcoding does further enable detection of named taxa, some likely of concern in biosecurity contexts, additional quality assurance steps are required to establish confidence in such detections. The utility of these approaches is likely to grow in coming years with rapid improvements in reference databases, sequencing technology, bioinformatic processing, and understanding of the ecology of environmental DNA (e.g. its persistence after the death of source organisms).

## Supplementary Material

Supplement1

## Figures and Tables

**Table 1:** Estimated number of vessels that would need to be sampled to achieve 50%, 90%, 95%, and 99% completeness of the theoretical OTU pool for each recipient port.

	Sample completeness			
	
Recipient port	50%	90%	95%	99%
AK	2	13	23	68
CB	6	93^a^	146^a^	270^a^
TX	5	63	99^a^	185^a^

a Maximum size of extrapolation exceeds double the reference sample size; results may be subject to large prediction bias.

## HIGHLIGHTS

- Metabarcoding enables comprehensive characterization of ballast water communities.
- Use of the 18S genomic locus allows assessment of dark and hidden diversity.
- Accumulation of diversity in recipient ports depends on the biogeography of ballast sources.
- In some cases it may take well over 100 vessels to saturate the species pool being delivered.
- Species of potential concern can be detected, but not without uncertainty.
