# Supplementary material for: Metabarcoding quantifies differences in accumulation of ballast water borne biodiversity among three port systems in the United States
Source: Sci Total Environ. Author manuscript; Available in PMC 2021 Dec 20. (PMC8190815; doi:10.1016/j.scitotenv.2020.141456)
Supplement: Supplement1 [file NIHMS1623217-supplement-Supplement1.docx]

**TITLE:** Metabarcoding reveals differences in the accumulation of ballast water borne biodiversity delivered to three port systems in the United States

**AUTHORS:** John A. Darling, John Martinson, Katrina Pagenkopp-Lohan, Katharine J. Carney, Erik Pilgrim, Aabir Banerji, Kimberly K. Holzer, Gregory M. Ruiz

# Supplemental Figures and Tables


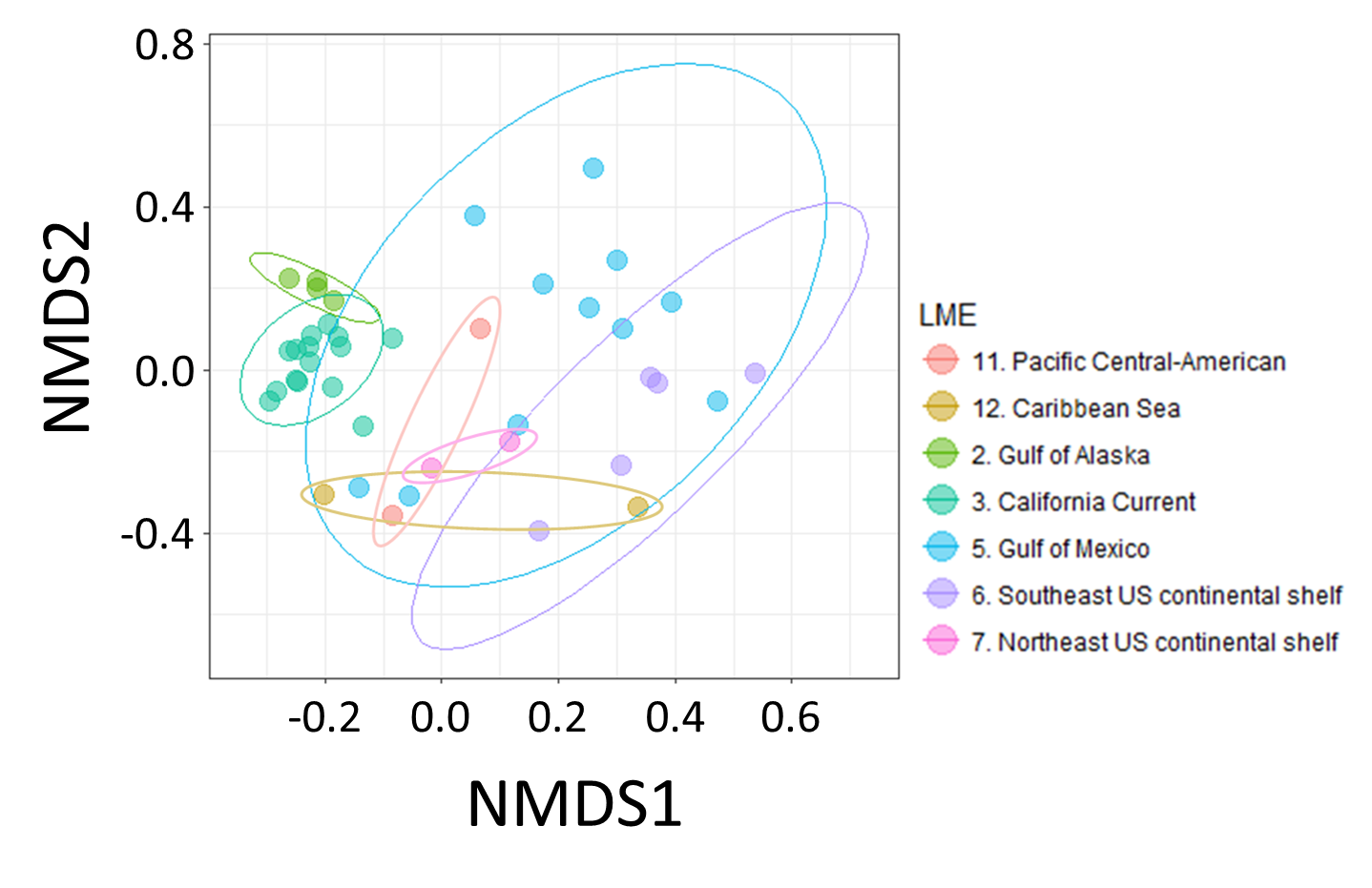


**Figure S1.** NMDS ordination of all samples from vessels entering AK and TX that have not conducted ballast water exchange. Clustering of vessels by source LME is significant (P < 0.001), but this is driven entirely by samples from AK (shown in green); if we only look at vessels entering TX, there is no significant effect of source LME on the ordination (P = 0.1849). Vessels entering CB were not included in the analysis as so few failed to conduct BWE.


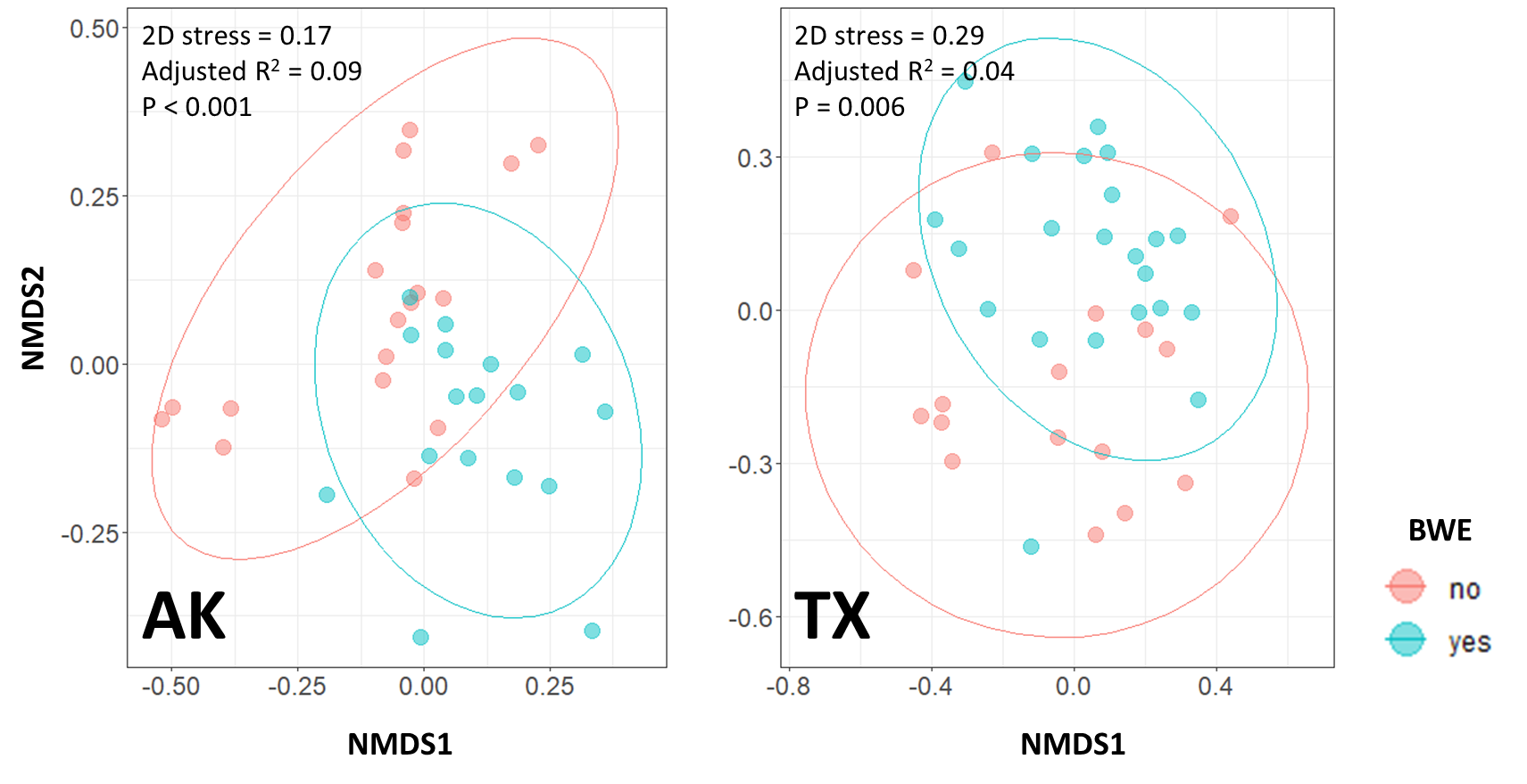


**Figure S2.** Clustering of vessels based on management status. Weak but significant separation of vessels conducting BWE and those not conducted BWE is observed for both AK and TX arrivals. CB arrivals were not analyzed as almost all vessels conducted BWE.


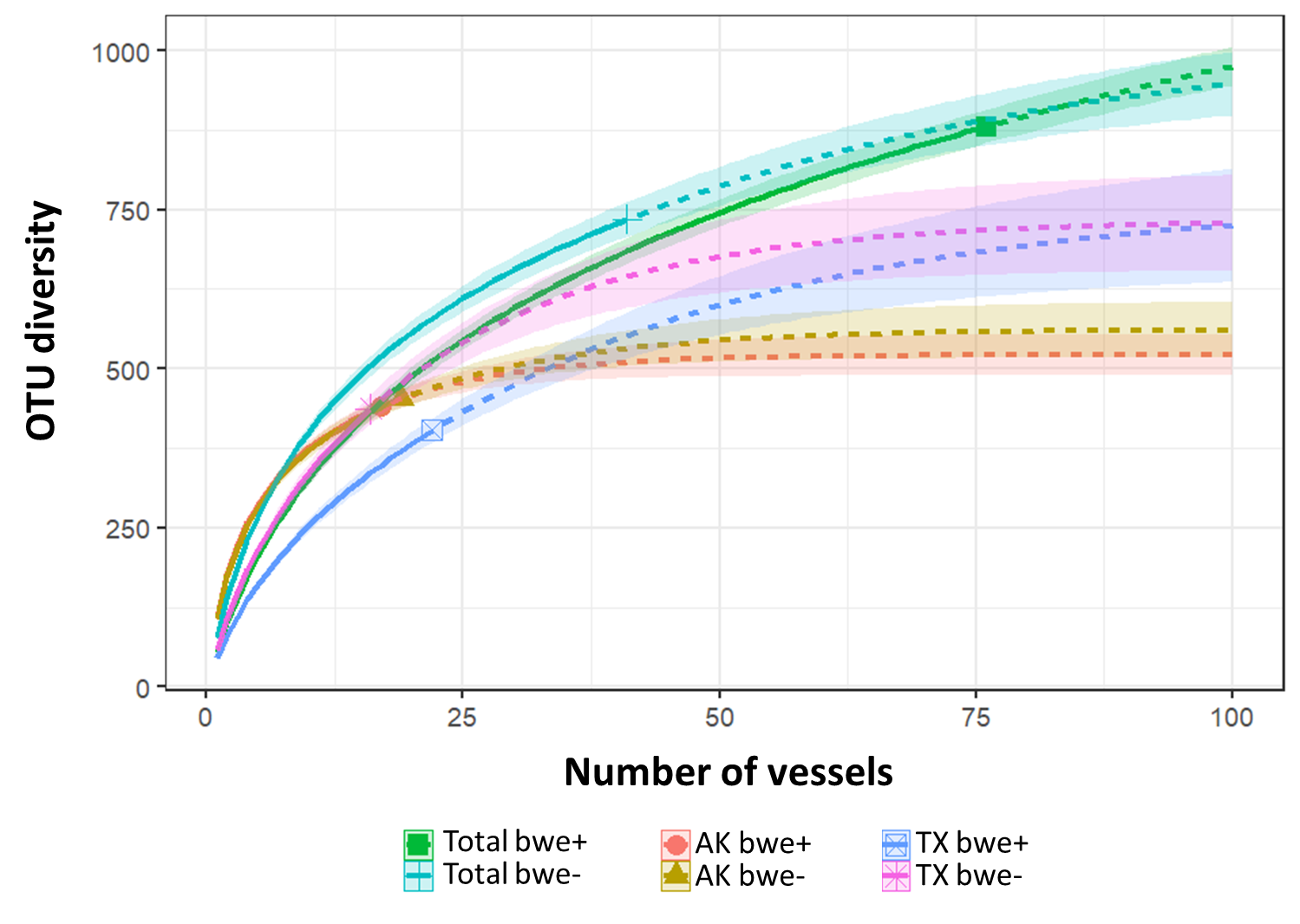


**Figure S3.** OTU accumulation curves for managed and unmanaged vessels. Curves are shown for total vessels as well as for AK and TX; CB vessels are not broken out by management category because almost all vessels entering CB undergo BWE. Solid lines show interpolation based on sampled vessels; dashed lines show extrapolation.

**Table S1.** Summary of samples.

| **Sample ID** | **Destination Port** | **Source Port** | **Source Country** | **BWE** | **Source LME** | **Voyage Length** | **BW Age** | **Source Latitude** | **Source Longitude** | **BWE Latitude** | **BWE Longitude** | **Read count** | **OTU count** | **Chao’s estimated OTU richness** | **Shannon Diversity** |
| --- | --- | --- | --- | --- | --- | --- | --- | --- | --- | --- | --- | --- | --- | --- | --- |
| VMT21 | AK | Nikiski | USA | no | 2. Gulf of Alaska | 2 | 2 | 60.68 | -151.39 | na | na | 26596 | 54 | 73.13 | 1.18 |
| VMT25 | AK | Martinez | USA | no | 3. California Current | 7 | 7 | 38.03 | -122.13 | na | na | 15183 | 112 | 130.45 | 1.76 |
| VMT30 | AK | Anacortes | USA | no | 3. California Current | 10 | 10 | 48.52 | -122.62 | na | na | 27575 | 80 | 123.50 | 0.59 |
| VMT33 | AK | Richmond | USA | no | 3. California Current | 7 | 7 | 37.92 | -122.38 | na | na | 13848 | 148 | 177.06 | 2.86 |
| VMT34 | AK | Nikiski | USA | no | 2. Gulf of Alaska | 2 | 2 | 60.68 | -151.39 | na | na | 34564 | 74 | 91.77 | 1.60 |
| VMT41 | AK | Ferndale | USA | no | 3. California Current | 5 | 5 | 48.83 | -122.72 | na | na | 26775 | 156 | 200.40 | 2.41 |
| VMT44 | AK | Nikiski | USA | no | 2. Gulf of Alaska | 3 | 3 | 60.55 | -151.22 | na | na | 33128 | 59 | 72.60 | 1.40 |
| VMT45 | AK | Richmond | USA | no | 3. California Current | 8 | 8 | 37.92 | -122.38 | na | na | 24964 | 115 | 137.89 | 1.16 |
| VMT46 | AK | Offshore Oregon VMT46 | USA | no | 3. California Current | 6 | 6 | 44.12 | -125.90 | na | na | 10517 | 115 | 158.33 | 1.87 |
| VMT47 | AK | Anacortes | USA | no | 3. California Current | 9 | 9 | 48.52 | -122.62 | na | na | 21750 | 172 | 207.05 | 2.91 |
| VMT48 | AK | Kenai | USA | no | 2. Gulf of Alaska | 2 | 2 | 60.55 | -151.22 | na | na | 44459 | 77 | 92.00 | 1.55 |
| VMT49 | AK | Richmond | USA | no | 3. California Current | 6 | 6 | 37.92 | -122.38 | na | na | 33670 | 122 | 131.23 | 2.76 |
| VMT50 | AK | Offshore Oregon VMT50 | USA | no | 3. California Current | 17 | 17 | 44.70 | -126.50 | na | na | 26194 | 62 | 65.50 | 1.34 |
| VMT56 | AK | Anacortes | USA | no | 3. California Current | 4 | 4 | 48.52 | -122.62 | na | na | 23027 | 138 | 201.91 | 2.45 |
| VMT57 | AK | Long Beach | USA | no | 3. California Current | 7 | 7 | 33.75 | -118.22 | na | na | 36576 | 129 | 239.63 | 0.42 |
| VMT61 | AK | Anacortes | USA | no | 3. California Current | 4 | 4 | 48.52 | -122.62 | na | na | 12030 | 91 | 124.21 | 1.37 |
| VMT65 | AK | Los Angeles | USA | no | 3. California Current | 9 | 9 | 33.75 | -118.23 | na | na | 22155 | 91 | 114.40 | 1.13 |
| VMT66 | AK | Anacortes | USA | no | 3. California Current | 7 | 7 | 48.52 | -122.62 | na | na | 12762 | 107 | 144.19 | 2.10 |
| VMT67 | AK | Ferndale | USA | no | 3. California Current | 5 | 5 | 48.83 | -122.72 | na | na | 10942 | 146 | 167.37 | 2.70 |
| CB19 | CB | Charleston | USA | no | 6. Southeast US continental shelf | 11 | 11 | 32.78 | -79.93 | na | na | 35995 | 27 | 79.50 | 0.99 |
| CB26 | CB | Somerset | USA | no | 7. Northeast US continental shelf | 7 | 7 | 41.77 | -71.13 | na | na | 49851 | 58 | 79.86 | 1.13 |
| CB28 | CB | Mobile | USA | no | 5. Gulf of Mexico | 16 | 16 | 30.68 | -88.05 | na | na | 45769 | 39 | 48.00 | 0.35 |
| CB29 | CB | Mobile | USA | no | 5. Gulf of Mexico | 28 | 28 | 30.68 | -88.05 | na | na | 23686 | 32 | 45.75 | 1.95 |
| CB50 | CB | Charleston | USA | no | 6. Southeast US continental shelf | 6 | 6 | 32.78 | -79.93 | na | na | 48610 | 32 | 48.50 | 0.87 |
| CB57 | CB | Bridgeport Anchorage | USA | no | 7. Northeast US continental shelf | 9 | 9 | 41.15 | -73.18 | na | na | 56750 | 47 | 71.00 | 0.20 |
| TXC03 | TX | Jacksonville | USA | no | 6. Southeast US continental shelf | 7 | 7 | 30.32 | -81.65 | na | na | 24383 | 39 | 41.00 | 2.22 |
| TXC06 | TX | Offshore Coatzacoalcos TXC06 | Mexico | no | 5. Gulf of Mexico | 8 | 8 | 21.98 | -94.42 | na | na | 27425 | 57 | 64.00 | 2.72 |
| TXC12 | TX | Houston | USA | no | 5. Gulf of Mexico | 8 | 8 | 29.75 | -95.33 | na | na | 28336 | 64 | 67.33 | 1.75 |
| TXC14 | TX | New Orleans | USA | no | 5. Gulf of Mexico | 3 | 3 | 29.95 | -90.06 | na | na | 36654 | 42 | 77.00 | 1.73 |
| TXC19 | TX | Tuxpan | Mexico | no | 5. Gulf of Mexico | 9 | 9 | 20.98 | -97.33 | na | na | 33281 | 56 | 61.00 | 3.27 |
| TXC21 | TX | Grand View Anchorage | USA | no | 5. Gulf of Mexico | 4 | 4 | 30.05 | -90.50 | na | na | 16235 | 20 | 27.50 | 1.92 |
| TXC26 | TX | Houston | USA | no | 5. Gulf of Mexico | 1 | 1 | 29.75 | -95.33 | na | na | 18641 | 38 | 38.60 | 1.49 |
| TXC28 | TX | Savannah | USA | no | 6. Southeast US continental shelf | 9 | 9 | 32.08 | -81.08 | na | na | 26812 | 21 | 25.20 | 0.02 |
| TXC29 | TX | Jacksonville | USA | no | 6. Southeast US continental shelf | 10 | 10 | 32.08 | -81.08 | na | na | 42162 | 44 | 57.20 | 0.72 |
| TXC32 | TX | Pajaritos | Mexico | no | 12. Caribbean Sea | 10 | 10 | 18.13 | -94.41 | na | na | 31375 | 27 | 54.50 | 1.10 |
| TXC37 | TX | Gulf of Mexico A | USA | no | 5. Gulf of Mexico | 21 | 21 | 26.00 | -90.65 | na | na | 19192 | 43 | 54.25 | 1.88 |
| TXC39 | TX | Pozos Colorados | Colombia | no | 12. Caribbean Sea | 6 | 6 | 11.15 | 74.25 | na | na | 26943 | 71 | 76.63 | 2.07 |
| TXC41 | TX | Veracruz | Mexico | no | 5. Gulf of Mexico | 6 | 6 | 19.20 | -96.13 | na | na | 35530 | 97 | 108.67 | 2.52 |
| TXC47 | TX | Las Minas | Panama | no | 11. Pacific Central-American | 9 | 9 | 9.40 | -79.83 | na | na | 28975 | 107 | 115.08 | 1.76 |
| TXC49 | TX | Tuxpan | Mexico | no | 5. Gulf of Mexico | 6 | 6 | 20.98 | -97.33 | na | na | 34736 | 136 | 146.00 | 1.63 |
| TXC51 | TX | Puerto Quetzal | Guatemala | no | 11. Pacific Central-American | 10 | 10 | 13.92 | -90.78 | na | na | 36147 | 59 | 77.20 | 2.29 |
| VMT20 | AK | Long Beach | USA | yes | 3. California Current | 22 | 3 | 33.75 | -118.22 | 49.17 | -134.15 | 47439 | 129 | 187.00 | 2.02 |
| VMT22 | AK | Long Beach | USA | yes | 3. California Current | 10 | 8 | 33.75 | -118.22 | 38.08 | -125.38 | 8461 | 56 | 60.50 | 1.60 |
| VMT24 | AK | Ferndale | USA | yes | 3. California Current | 9 | 2 | 48.83 | -122.72 | 53.49 | -138.97 | 19853 | 119 | 128.24 | 2.14 |
| VMT27 | AK | Long Beach | USA | yes | 3. California Current | 10 | 6 | 33.75 | -118.22 | 46.00 | -125.95 | 9321 | 101 | 139.15 | 1.28 |
| VMT29 | AK | Anacortes | USA | yes | 3. California Current | 10 | 2 | 48.52 | -122.62 | 52.37 | -136.28 | 17226 | 146 | 164.21 | 1.65 |
| VMT31 | AK | Benicia | USA | yes | 3. California Current | 6 | 2 | 38.04 | -122.14 | 51.92 | -136.77 | 22464 | 57 | 70.60 | 1.90 |
| VMT35 | AK | Cherry Point | USA | yes | 3. California Current | 4 | 1 | 48.86 | -122.76 | 55.72 | -142.45 | 8746 | 41 | 60.50 | 2.19 |
| VMT37 | AK | Benicia | USA | yes | 3. California Current | 5 | 4 | 38.04 | -122.14 | 41.92 | -127.12 | 27319 | 125 | 162.06 | 1.92 |
| VMT39 | AK | Anacortes | USA | yes | 3. California Current | 5 | 2 | 48.52 | -122.62 | 51.53 | -134.87 | 27595 | 162 | 186.70 | 2.15 |
| VMT40 | AK | Ferndale | USA | yes | 3. California Current | 5 | 3 | 48.83 | -122.72 | 51.54 | -133.53 | 10103 | 125 | 148.00 | 3.14 |
| VMT42 | AK | Cherry Point | USA | yes | 3. California Current | 4 | 3 | 48.86 | -122.76 | 51.03 | -133.90 | 19329 | 136 | 178.50 | 1.71 |
| VMT54 | AK | Cherry Point | USA | yes | 3. California Current | 4 | 3 | 48.86 | -122.76 | 50.28 | -131.11 | 24204 | 157 | 194.27 | 2.07 |
| VMT55 | AK | Long Beach | USA | yes | 3. California Current | 8 | 5 | 33.75 | -118.22 | 47.79 | -125.93 | 35426 | 72 | 97.67 | 0.50 |
| VMT62 | AK | Anacortes | USA | yes | 3. California Current | 4 | 2 | 48.52 | -122.62 | 51.71 | -134.48 | 27704 | 144 | 189.88 | 1.85 |
| VMT63 | AK | Cherry Point | USA | yes | 3. California Current | 5 | 2 | 48.86 | -122.76 | 53.92 | -134.28 | 16728 | 115 | 172.27 | 2.21 |
| VMT64 | AK | Anacortes | USA | yes | 3. California Current | 6 | 2 | 48.52 | -122.62 | 53.93 | -138.58 | 12850 | 98 | 120.14 | 1.71 |
| VMT68 | AK | Cherry Point | USA | yes | 3. California Current | 6 | 4 | 48.86 | -122.76 | 49.87 | -130.12 | 20379 | 73 | 95.24 | 0.79 |
| CB02 | CB | Amsterdam | Netherlands | yes | 22. North Sea | 14 | 6 | 52.37 | 4.90 | 43.13 | -42.43 | 31778 | 39 | 44.60 | 1.37 |
| CB06 | CB | Canso | Canada | yes | 8. Scotian Shelf | 8 | 6 | 45.34 | -61.00 | 41.91 | -64.00 | 17575 | 21 | 26.25 | 0.26 |
| CB08 | CB | Coastal Uribia | Colombia | yes | 12. Caribbean Sea | 18 | 4 | 13.83 | -72.73 | 29.52 | -72.07 | 43808 | 108 | 127.00 | 2.00 |
| CB12 | CB | Rotterdam | Netherlands | yes | 22. North Sea | 24 | 11 | 51.90 | 4.44 | 44.53 | -37.13 | 37429 | 15 | 21.00 | 1.17 |
| CB13 | CB | Rotterdam | Netherlands | yes | 22. North Sea | 15 | 9 | 51.90 | 4.44 | 51.18 | -26.12 | 51064 | 82 | 95.00 | 2.27 |
| CB14 | CB | Port Alfred | Canada | yes | 9. Newfoundland-Labrador Shelf | 6 | 2 | 48.33 | 70.87 | 41.23 | -63.33 | 19682 | 25 | 34.33 | 1.25 |
| CB16 | CB | Belfast | Ireland | yes | 24. Celtic-Biscay Shelf | 16 | 11 | 54.60 | -5.93 | 53.13 | -24.42 | 40705 | 13 | 23.00 | 1.12 |
| CB17 | CB | Rotterdam | Netherlands | yes | 22. North Sea | 29 | 20 | 51.90 | 4.44 | 48.40 | -23.56 | 25542 | 40 | 43.33 | 2.59 |
| CB18 | CB | Money Point | Ireland | yes | 24. Celtic-Biscay Shelf | 19 | 14 | 52.60 | -9.42 | 52.23 | -21.48 | 13200 | 24 | 31.50 | 2.44 |
| CB20 | CB | Amsterdam | Netherlands | yes | 22. North Sea | 18 | 13 | 52.37 | 4.90 | 49.38 | -31.29 | 47957 | 38 | 53.00 | 0.75 |
| CB21 | CB | Canso | Canada | yes | 8. Scotian Shelf | 3 | 2 | 45.34 | -61.00 | 41.23 | -65.20 | 53393 | 44 | 47.75 | 1.75 |
| CB23 | CB | Hamburg | Germany | yes | 22. North Sea | 15 | 6 | 53.55 | 9.97 | 43.80 | -42.99 | 25579 | 41 | 56.60 | 1.53 |
| CB24 | CB | Ghent | Belgium | yes | 22. North Sea | 17 | 10 | 51.07 | 3.72 | 49.65 | -14.87 | 18406 | 20 | 34.00 | 1.58 |
| CB25 | CB | Port Alfred | Canada | yes | 9. Newfoundland-Labrador Shelf | 8 | 4 | 48.33 | 70.87 | 38.65 | -59.83 | 16763 | 15 | 17.00 | 1.84 |
| CB27 | CB | Stade | Germany | yes | 22. North Sea | 21 | 13 | 53.60 | 9.05 | 50.47 | -22.25 | 38465 | 30 | 44.00 | 1.68 |
| CB30 | CB | Aughinish Island | Ireland | yes | 24. Celtic-Biscay Shelf | 15 | 10 | 52.58 | -9.68 | 45.88 | -32.19 | 23896 | 128 | 130.81 | 2.56 |
| CB31 | CB | Aughinish Island | Ireland | yes | 24. Celtic-Biscay Shelf | 14 | 8 | 52.58 | -9.68 | 51.58 | -27.08 | 38466 | 42 | 51.33 | 1.94 |
| CB32 | CB | Brindisi | Italy | yes | 26. Mediterranean | 38 | 26 | 40.65 | 17.98 | 41.35 | -36.53 | 20099 | 43 | 78.00 | 2.25 |
| CB33 | CB | Redcar | United Kingdom | yes | 22. North Sea | 16 | 7 | 54.62 | -1.15 | 50.09 | -34.40 | 45307 | 45 | 56.00 | 1.61 |
| CB34 | CB | Port Alfred | Canada | yes | 9. Newfoundland-Labrador Shelf | 6 | 1 | 48.33 | 70.87 | 42.93 | -60.22 | 10789 | 26 | 27.50 | 1.86 |
| CB38 | CB | Ijmuiden | Netherlands | yes | 22. North Sea | 14 | 9 | 52.46 | 4.58 | 46.92 | -23.43 | 13800 | 19 | 37.00 | 1.53 |
| CB39 | CB | Rotterdam | Netherlands | yes | 22. North Sea | 14 | 10 | 51.90 | 4.44 | 47.20 | -18.39 | 31574 | 6 | 7.00 | 0.00 |
| CB40 | CB | Montoir | France | yes | 25. Iberian Coastal | 21 | 13 | 47.33 | -2.13 | 44.82 | -19.76 | 30767 | 18 | 22.20 | 0.02 |
| CB43 | CB | Rotterdam | Netherlands | yes | 22. North Sea | 17 | 10 | 51.90 | 4.44 | 42.02 | -39.51 | 42562 | 20 | 65.50 | 0.03 |
| CB44 | CB | Port Alfred | Canada | yes | 9. Newfoundland-Labrador Shelf | 12 | 7 | 48.33 | 70.87 | 39.27 | -59.71 | 13889 | 34 | 36.00 | 2.37 |
| CB45 | CB | Amsterdam | Netherlands | yes | 22. North Sea | 35 | 24 | 52.37 | 4.90 | 48.07 | -12.23 | 15744 | 31 | 57.25 | 2.25 |
| CB46 | CB | Rotterdam | Netherlands | yes | 22. North Sea | 30 | 16 | 51.90 | 4.44 | 41.93 | -41.82 | 19824 | 38 | 44.43 | 2.54 |
| CB47 | CB | Gijon | Spain | yes | 25. Iberian Coastal | 17 | 9 | 43.55 | -5.67 | 42.15 | -41.40 | 16448 | 33 | 51.33 | 2.24 |
| CB48 | CB | Carboneras | Spain | yes | 26. Mediterranean | 15 | 6 | 37.00 | -1.88 | 30.50 | -41.50 | 36434 | 65 | 68.00 | 2.42 |
| CB49 | CB | Iskenderun | Turkey | yes | 26. Mediterranean | 30 | 16 | 36.59 | 36.18 | 40.83 | -38.82 | 11495 | 31 | 38.00 | 2.26 |
| CB51 | CB | Fusina | Italy | yes | 26. Mediterranean | 20 | 6 | 45.42 | 12.25 | 40.82 | -39.70 | 9968 | 21 | 28.50 | 0.40 |
| CB52 | CB | Civitavecchia | Italy | yes | 26. Mediterranean | 30 | 6 | 42.10 | 11.80 | 40.85 | -40.22 | 14092 | 32 | 37.60 | 2.32 |
| CB53 | CB | Ghent | Belgium | yes | 22. North Sea | 28 | 23 | 51.07 | 3.72 | 48.08 | -15.07 | 13586 | 34 | 35.00 | 2.56 |
| CB54 | CB | Cartagena | Colombia | yes | 12. Caribbean Sea | 30 | 14 | 10.38 | -75.50 | 34.99 | -70.75 | 18728 | 18 | 33.00 | 0.78 |
| CB55 | CB | Redcar | United Kingdom | yes | 22. North Sea | 30 | 20 | 54.62 | -1.15 | 44.22 | -33.87 | 42877 | 19 | 21.00 | 0.02 |
| CB56 | CB | Isdemir | Turkey | yes | 26. Mediterranean | 24 | 9 | 36.59 | 36.18 | 36.36 | -35.31 | 34422 | 52 | 61.17 | 1.31 |
| TXC05 | TX | Tuxpan | Mexico | yes | 5. Gulf of Mexico | 2 | 1 | 20.98 | -97.33 | 23.41 | -95.90 | 33693 | 125 | 128.00 | 3.03 |
| TXC07 | TX | Savannah | USA | yes | 6. Southeast US continental shelf | 7 | 4 | 32.08 | -81.08 | 25.58 | -88.78 | 33433 | 25 | 36.00 | 0.13 |
| TXC08 | TX | Rosarito | Mexico | yes | 11. Pacific Central-American | 18 | 11 | 32.37 | -117.08 | 9.58 | -90.42 | 32931 | 28 | 32.20 | 0.57 |
| TXC09 | TX | Esmeraldas | Ecuador | yes | 11. Pacific Central-American | 13 | 6 | 0.97 | -79.68 | 14.00 | -79.67 | 38585 | 44 | 57.75 | 2.72 |
| TXC17 | TX | Mejillones | Chile | yes | 13. Humboldt Current | 25 | 22 | -23.10 | -70.47 | 12.86 | -81.58 | 17877 | 16 | 17.50 | 1.50 |
| TXC20 | TX | Tarragona | Spain | yes | 26. Mediterranean | 48 | 14 | 41.10 | 1.23 | 44.63 | -46.05 | 15865 | 28 | 37.00 | 2.09 |
| TXC24 | TX | Callao | Peru | yes | 13. Humboldt Current | 35 | 7 | -12.05 | -77.15 | 18.42 | -81.80 | 20012 | 24 | 36.00 | 1.46 |
| TXC25 | TX | Callao | Peru | yes | 13. Humboldt Current | 36 | 8 | -12.05 | -77.15 | 15.17 | -79.63 | 9365 | 16 | 30.00 | 1.06 |
| TXC30 | TX | Pozos Colorados | Colombia | yes | 12. Caribbean Sea | 6 | 4 | 11.15 | 74.25 | 13.07 | -75.83 | 41392 | 54 | 55.43 | 1.39 |
| TXC31 | TX | Pozos Colorados | Colombia | yes | 12. Caribbean Sea | 6 | 4 | 11.15 | 74.25 | 18.17 | -81.55 | 30223 | 30 | 60.60 | 0.04 |
| TXC33 | TX | Tuxpan | Mexico | yes | 5. Gulf of Mexico | 12 | 7 | 20.98 | -97.33 | 23.68 | -93.82 | 26048 | 36 | 46.50 | 1.58 |
| TXC34 | TX | Sao Luis | Brazil | yes | 17. North Brazil Shelf | 21 | 20 | -2.52 | -44.27 | 2.76 | -47.15 | 38540 | 63 | 87.43 | 2.73 |
| TXC35 | TX | Sao Luis | Brazil | yes | 17. North Brazil Shelf | 22 | 18 | -2.52 | -44.27 | 6.41 | -52.29 | 31645 | 46 | 72.00 | 2.39 |
| TXC38 | TX | Omoa | Honduras | yes | 12. Caribbean Sea | 9 | 1 | 15.77 | -88.03 | 25.88 | -89.90 | 22205 | 33 | 46.75 | 1.92 |
| TXC42 | TX | Sao Sebastiao | Brazil | yes | 15. South Brazil shelf | 20 | 13 | -2.52 | -44.27 | 2.84 | -45.96 | 18664 | 66 | 73.50 | 3.22 |
| TXC43 | TX | Offshore Nova Scotia TXC44 | Canada | yes | 8. Scotian Shelf | 22 | 11 | 44.67 | -63.57 | 38.31 | -67.32 | 27268 | 55 | 66.00 | 1.81 |
| TXC44 | TX | Dartmouth | Canada | yes | 8. Scotian Shelf | 12 | 9 | 44.67 | -63.57 | 29.91 | -74.92 | 19628 | 50 | 59.33 | 2.51 |
| TXC45 | TX | Arica | Chile | yes | 13. Humboldt Current | 21 | 6 | -18.48 | -70.33 | 19.53 | -83.58 | 28119 | 54 | 62.25 | 0.83 |
| TXC48 | TX | Tees | United Kingdom | yes | 22. North Sea | 19 | 7 | 54.60 | -1.17 | 33.20 | -56.56 | 17827 | 34 | 45.25 | 1.99 |
| TXC50 | TX | Puerto Moin | Costa Rica | yes | 12. Caribbean Sea | 6 | 4 | 10.00 | -83.08 | 19.61 | 83.20 | 43197 | 57 | 88.50 | 0.84 |
| TXC52 | TX | Quintero | Chile | yes | 13. Humboldt Current | 17 | 11 | 32.77 | -71.50 | -7.00 | -81.22 | 41859 | 49 | 52.75 | 3.18 |
| TXC53 | TX | Quintero | Chile | yes | 13. Humboldt Current | 17 | 12 | 32.77 | -71.50 | -11.99 | -79.51 | 26998 | 52 | 61.33 | 2.48 |

**Table S2.** OTUs assigned to species recognized as invasive, either in the United States (NEMESIS = “yes”) or globally. Notes are given on BLAST searches conducted to assess confidence in initial taxonomic assignments. Counts are uncorrected sequence counts.

| **Phylum** | **Species** | **RDP score** | **Total Count** | **AK count (# vessels)** | **CB count (# vessels)** | **TX count (# vessels)** | **NEMESIS?** | **OTU #** | **BLAST results** |
| --- | --- | --- | --- | --- | --- | --- | --- | --- | --- |
| Annelida | *Alitta succinea* | 1.00 | 16732 | 9 (4) | 16721 (3) | 2 (1) | yes | OTU53 | top hit to *Nereis succinea* (100%), synonym for *Alitta succinea* |
| Annelida | *Pseudopolydora paucibranchiata* | 1.00 | 804 | 802 (16) | 0 (0) | 2 (1) | yes | OTU285 | top hit to *P. paucibranchiata* (100%) |
| Arthropoda | *Acartia tonsa* | 1.00 | 153 | 0 (0) | 0 (0) | 153 (1) |  | OTU945 | all top hits to *A. tonsa* (97-99%) |
| Arthropoda | *Eurytemora pacifica* | 1.00 | 19 | 19 (1) | 0 (0) | 0 (0) |  | OTU1093 | *E. pacifica* is top hit (99%), next highest is 90% |
| Arthropoda | *Eurytemora pacifica* | 0.89 | 2 | 2 (1) | 0 (0) | 0 (0) |  | OTU986 | all top hits to uncultured eukaryote, top match 92% |
| Arthropoda | *Neomysis integer* | 0.87 | 144 | 125 (1) | 0 (0) | 19 (2) |  | OTU862 | *N. integer* top hit (97%) and 4 of top 5; *N. americana* 2nd hit also at 97% |
| Arthropoda | *Neomysis integer* | 0.71 | 50672 | 16292 (8) | 465 (9) | 33915 (14) |  | OTU19 | *N. integer* top hit (100%) and 4 of top 5; *N. americana* 2nd hit also at 100% |
| Bacillariophyta | *Ditylum brightwellii* | 1.00 | 41 | 0 (0) | 1 (1) | 40 (1) |  | OTU825 | top 7 hits to *D. brightwellii* (95-99%), next highest at 87% |
| Bryozoa | *Bugulina stolonifera* | 0.54 | 87 | 70 (2) | 17 (1) | 0 (0) | yes | OTU615 | top three hits to *Scruparia chelata*, *Bugula stolonifera*, and *Electra pilosa* (all 95%) |
| Bryozoa | *Membranipora membranacea* | 0.92 | 564 | 564 (17) | 0 (0) | 0 (0) | yes | OTU415 | top two hits to *M. membranacea* (99%), next highest at 96% |
| Cnidaria | *Diadumene leucolena* | 0.96 | 350 | 13 (2) | 59 (2) | 278 (1) | yes | OTU490 | top hit *D. leucolena* (100%), next highest at 98% |
| Cnidaria | *Muggiaea atlantica* | 0.64 | 1444 | 9 (2) | 0 (0) | 1435 (3) |  | OTU162 | most top hits uncultured eukaryote; top names hit *M. atlantica* (99%); *M. sp* and *Spaeronectes christiansonae* also 99% |
| Cnidaria | *Obelia dichotoma* | 0.66 | 457 | 431 (24) | 0 (0) | 26 (1) |  | OTU336 | *O. dichomata* top six hits (100%); next hit *O. geniculata* (99%) |
| Miozoa | *Alexandrium catenella* | 0.61 | 0 | 0 (0) | 0 (0) | 0 (0) |  | OTU1134 | top hits to uncultured eukaryote (99%); multiple *Alexandrium sp* all at 97% |
| Miozoa | *Alexandrium monilatum* | 1.00 | 9 | 0 (0) | 9 (1) | 0 (0) |  | OTU768 | *A. monilatum* is top hit (100%), next highest is 92% |
| Miozoa | *Ceratoperidinium falcatum* | 0.90 | 98 | 2 (1) | 51 (1) | 45 (1) |  | OTU609 | top two hits to *C. falcatum* (99%), other top hits to uncultured eukaryotes |
| Miozoa | *Dinophysis caudata* | 1.00 | 509 | 100 (15) | 52 (3) | 357 (2) |  | OTU312 | top hits to other *Dinophysis* species at 100%, *D. caudata* at 98% |
| Miozoa | *Dinophysis caudata* | 1.00 | 24 | 0 (0) | 0 (0) | 24 (1) |  | OTU572 | top hits to other *Dinophysis* species at 100%, *D. caudata* at 98% |
| Miozoa | *Dinophysis caudata* | 1.00 | 5 | 0 (0) | 5 (1) | 0 (0) |  | OTU1043 | top hits to other *Dinophysis* species at 100%, *D. caudata* at 98% |
| Miozoa | *Karlodinium veneficum* | 0.97 | 598 | 101 (16) | 367 (5) | 130 (1) |  | OTU277 | all top hits to uncultured eukaryote; top named hit is *K. micrum* at 97% |
| Miozoa | *Karlodinium veneficum* | 1.00 | 53 | 7 (3) | 46 (2) | 0 (0) |  | OTU543 | all top hits to uncultured eukaryote; top named hit is *K. micrum* at 97% |
| Miozoa | *Karlodinium veneficum* | 1.00 | 40 | 18 (6) | 22 (1) | 0 (0) |  | OTU759 | all top hits to uncultured eukaryote; top named hit is *K. micrum* at 97% |
| Miozoa | *Levanderina fissa* | 0.91 | 22 | 22 (5) | 0 (0) | 0 (0) |  | OTU943 | *l. fissa* is second hit (99%), top hit is *Gyrodinium uncatenum*; *Gyrodinium sp* are 9 of top 10 hits |
| Miozoa | *Phalacroma mitra* | 0.87 | 13 | 5 (2) | 8 (3) | 0 (0) |  | OTU1126 | *P. mitra* top hit (99%); next hit 97%; other *Phalcroma sp.* In top 6 hits, all 96% or greater |
| Miozoa | *Phalacroma rotundatum* | 0.59 | 3 | 0 (0) | 3 (1) | 0 (0) |  | OTU957 | top hit to *Dinophysis rotundata* (96%); *P. oxytoxoides* second hit (96%); multiple *Phalacroma sp* in top 5 hits |
| Mollusca | *Geukensia demissa* | 0.67 | 7 | 0 (0) | 0 (0) | 7 (1) | yes | OTU1163 | *G. demissa* three of top 4 hits (97%); *B. exustus* second hit also at 97% |
| Mollusca | *Martesia striata* | 0.99 | 43 | 0 (0) | 0 (0) | 43 (2) |  | OTU885 | top hit to *M. striata* (100%), next highest at 98% |
| Mollusca | *Mytella charruana* | 0.94 | 430 | 0 (0) | 0 (0) | 430 (2) | yes | OTU341 | top hit *M. charruana* (100%), *Perna perna* at 99% |
| Mollusca | *Ruditapes (Venerupis) philippinarum* | 0.75 | 1706 | 1444 (23) | 1 (1) | 261 (3) | yes | OTU223 | *R. philippinarum* top 4 hits (100%), also 5th hit at 99% |
